# Knock-in *Kcnh2* rabbit model of long QT syndrome type-2, epilepsy, and sudden death

**DOI:** 10.1186/s12967-025-06382-w

**Published:** 2025-04-15

**Authors:** Veronica Singh, Kyle T. Wagner, Laura G. Williams, Justin M. Ryan, Katherine R. Keller, Jonathan D. Mohnkern, Robert S. Gardner, Louis T. Dang, Julie M. Ziobro, Richard J. H. Wojcikiewicz, Nathan R. Tucker, David S. Auerbach

**Affiliations:** 1 Department of Pharmacology, SUNY Upstate Medical University, Syracuse, NY USA; 2 Department of Pediatrics, University of Michigan, Ann Arbor, MI USA; 3 Department of Medicine-Cardiology, SUNY Upstate Medical University, Syracuse, NY USA; 4 Department of Pharmacology, Department of Medicine - Cardiology, SUNY Upstate Medical University, 750 East Adams St, Syracuse, NY 13210 USA

**Keywords:** Arrhythmia, Seizure, Epilepsy, Long QT syndrome, Rabbit, *KCNH2*, SUDEP

## Abstract

**Background:**

Long QT Syndrome Type-2 (LQT2) is due to loss-of-function *KCNH2* variants. *KCNH2* encodes K_v_11.1 that forms a delayed-rectifier potassium channel in the brain and heart. LQT2 is associated with arrhythmias, seizures, sudden cardiac death, and sudden unexpected death in epilepsy (SUDEP). The goal of the study is to develop a translational model that reproduces the neuro-cardiac electrical abnormalities and sudden death seen in people with LQT2.

**Methods:**

We generated the first knock-in rabbit model of LQT2 (*Kcnh2*^(+/7bp−del)^), due to a 7 base-pair (7bp) deletion in the pore domain of the endogenous rabbit *Kcnh2* gene.

**Results:**

Mutant *Kcnh2* is expressed in the heart and brain and constitutes 11% of total *Kcnh2* in *Kcnh2*^(+/7bp−del)^ rabbits. Total *Kcnh2*, WT *Kcnh2*, and WT K_v_11.1 expression is lower in *Kcnh2*^(+/7bp−del)^ vs. WT rabbits. *Kcnh2*^(+/7bp−del)^ rabbits exhibit prolonged cardiac ventricular repolarization (QT_c_, JT_ec_, JT_pc_). There is an increased prevalence of spontaneous epileptiform activity and clinical seizures in *Kcnh2*^(+/7bp−del)^ (7 of 37 rabbits) vs. WT rabbits (1:68 rabbits, *p* < 0.003). 18.9% of *Kcnh2*^(+/7bp−del)^ vs. 1.5% of WT rabbits died suddenly and spontaneously (*p* < 0.003). We recorded 2 spontaneous lethal events in *Kcnh2*^(+/7bp−del)^ rabbits: (1) sudden cardiac death and (2) seizure-mediated sudden death due to generalized tonic-clonic seizures, post-ictal generalized EEG suppression, bradycardia, ECG-T-wave inversion, focal cardiac activity, and asystole/death.

**Conclusions:**

We developed the first genetic rabbit model of LQT2 that reproduces the cardiac and epileptic phenotypes seen in people with LQT2. *Kcnh2*^(+/7bp−del)^ rabbits provide a valuable tool for future mechanistic studies, development of neurotherapeutics, and cardiac-safety testing.

**Supplementary Information:**

The online version contains supplementary material available at 10.1186/s12967-025-06382-w.

## Introduction

Long QT Syndrome (LQTS) is an ion channelopathy associated with a high risk of cardiac arrhythmias (e.g., *torsade de pointes*) and sudden cardiac death (SCD) [1]. It affects 1:2000 people and is characterized by prolongation of the cardiac electrical activation-recovery interval (QT_c_ on the electrocardiogram, ECG) [2, 3]. Long QT Syndrome Type-2 (LQT2) is due to loss-of-function (LOF) variants in the *KCNH2* gene [4, 5]. *KCNH2* encodes the K_v_11.1 protein, which forms the α-subunit of the channel that passes the rapid delayed rectifier potassium current (I_Kr_) [6]. I_Kr_ is responsible for cardiomyocyte repolarization [7] and suppression of repetitive action potential (AP) firing in neurons [8]. *KCNH2* LOF variants cause a reduction in I_Kr_, leading to cardiomyocyte AP prolongation and hyperexcitability, which ultimately provides a substrate for arrhythmias and SCD. Interestingly, there is a 3.7-fold higher prevalence of seizures in people genotype-positive for LQT2, compared to genotype-negative family members [9]. The prevalence of electroencephalogram (EEG) diagnosed epilepsy is higher in people with LQT2, compared to people with LQTS Type-1 (LQT1) and healthy controls without epilepsy [10, 11]. Post-mortem genetic analysis indicates a higher prevalence of LOF (3-fold) and rare (11-fold) *KCNH2* variants in Sudden Unexpected Death in Epilepsy (SUDEP) cases vs. living epilepsy controls [12]. Despite overwhelming evidence of a dual pathological role of *KCNH2* variants in the heart and brain, there is no translational model that fully reproduces the neuro-cardiac abnormalities seen in people with LQT2. There is an unmet need for a clinically relevant model of LQT2 to investigate the mechanisms for the high risk of seizures and SUDEP in LQT2 and *KCNH2*-mediated epilepsy.

The objective of this study is to generate and characterize a genetic rabbit model of *Kcnh2*-mediated epilepsy, ECG abnormalities, and sudden death (SCD & SUDEP), which reproduces the neuro-cardiac electrical abnormalities seen in people with LQT2. We developed the first knock-in rabbit model of LQT2 that is due to a heterozygous frameshift deletion mutation in the pore domain of the endogenous rabbit *Kcnh2* gene. *KCNH2* pore variants confer the highest risk of arrhythmias and seizures in people with LQT2 [1, 9]. A novel clinically relevant animal model of LQT2 will facilitate future studies to investigate the underlying mechanisms of EEG abnormalities, epileptic seizures, and SUDEP, as well as drug development and testing.

## Methods

All experiments were performed in accordance with the Guide for the Care and Use of Laboratory Animals and approved by the Institutional Animal Care and Use Committee.

### Generation of Knock-In rabbit model of LQT2:

Using CRISPR-Cas9 technology, the Center for Advanced Models and Translational Sciences and Therapeutics (CAMTraST) at the University of Michigan generated a founder rabbit (*Kcnh2*^(+/7bp−del)^) with a 7 bp frameshift deletion (1627–1633 bp, NM_001082384.1) in one allele of the endogenous rabbit *Kcnh2* gene. *Kcnh2*^(+/7bp−del)^ rabbits are cross-bred and maintained on the New Zealand White background (Charles River, Wilmington, Massachusetts). Rabbits > 1-month of age are housed in separate cages at 22ºC, fed *ad libitum*, and on a 12-hour light/dark cycle (lights on 6AM–6PM).

### RNA sequencing, Oxford Nanopore Technology (ONT) sequencing, and quantitative PCR (qPCR):

RNAseq libraries were constructed from rabbit left ventricle and brain stem samples using Zymo-Seq RiboFree Total RNA library kit and sequenced on an Illumina NovaseqX 10B flow cell. ONT sequencing was performed by Plasmidsaurus using primers that surround the mutation. For qPCR, PrimeTime probes (Integrated DNA Technologies) are designed using sequences specific to WT and mutant *Kcnh2* transcripts. Forward and reverse primers are also generated for beta-actin (Actb), which serves as the housekeeping gene. See supplement for details (Supplement Table 1).

### K_v_11.1 expression:

We verified the K_v_11.1 expression in HEK cells and rabbit tissue using commercially available antibodies (anti-MYC, anti-FLAG) and an antibody generated in-house (anti-K_v_11.1). See supplement for details.

### In-Vivo Video-ECG-EEG signal:

Cardiac (ECG), neuronal (EEG), audio, and video recordings are acquired from conscious restrained rabbits. Full details about the type of electrodes, EEG/ECG locations, and acquisition system are described in the supplement and in Bosinski et al. 2021 [13]. The electrode placements facilitate unipolar, bipolar, and referential EEG montages, as well as bipolar, augmented, and referential ECG configurations (Supplementary Fig. 1).

### ECG and EEG analysis:

All ECG data is analyzed using LabChart8 and average representative genotype-specific ECG traces are generated using MATLAB 2023b. From each rabbit, a 5-minute baseline period of ECG signal during a stable heart rate is analyzed. All video/EEG recordings are manually reviewed by an investigator blinded to the genotype and sex, and all epileptiform activity and seizures are confirmed by board-certified pediatric epileptologists (LTD & JMZ). A 60 Hz notch filter and 1–70 Hz bandpass filter is applied to all EEG signal.

### Statistical analysis:

Continuous data is tested for normality using Komogorov-Smirnov test. If normally distributed, t-test (for two groups) or ANOVA (for more than two groups) is used. If the data is not normally distributed, Mann-Whitney/Wilcoxon RankSum test (two groups) or Kruskal-Wallis (> 2 groups) is used. Logistic regression is used to adjust for multiple variables (age, sex, & heart rate) when testing for differences in ECG metrics and sudden death between groups (i.e., outcome measure). Chi-squared or Fisher’s exact test is used to test for differences between proportions. Mantel-Cox log-rank test is used for Kaplan-Meier survival curve analysis.

## Results

**Fig. 1:**
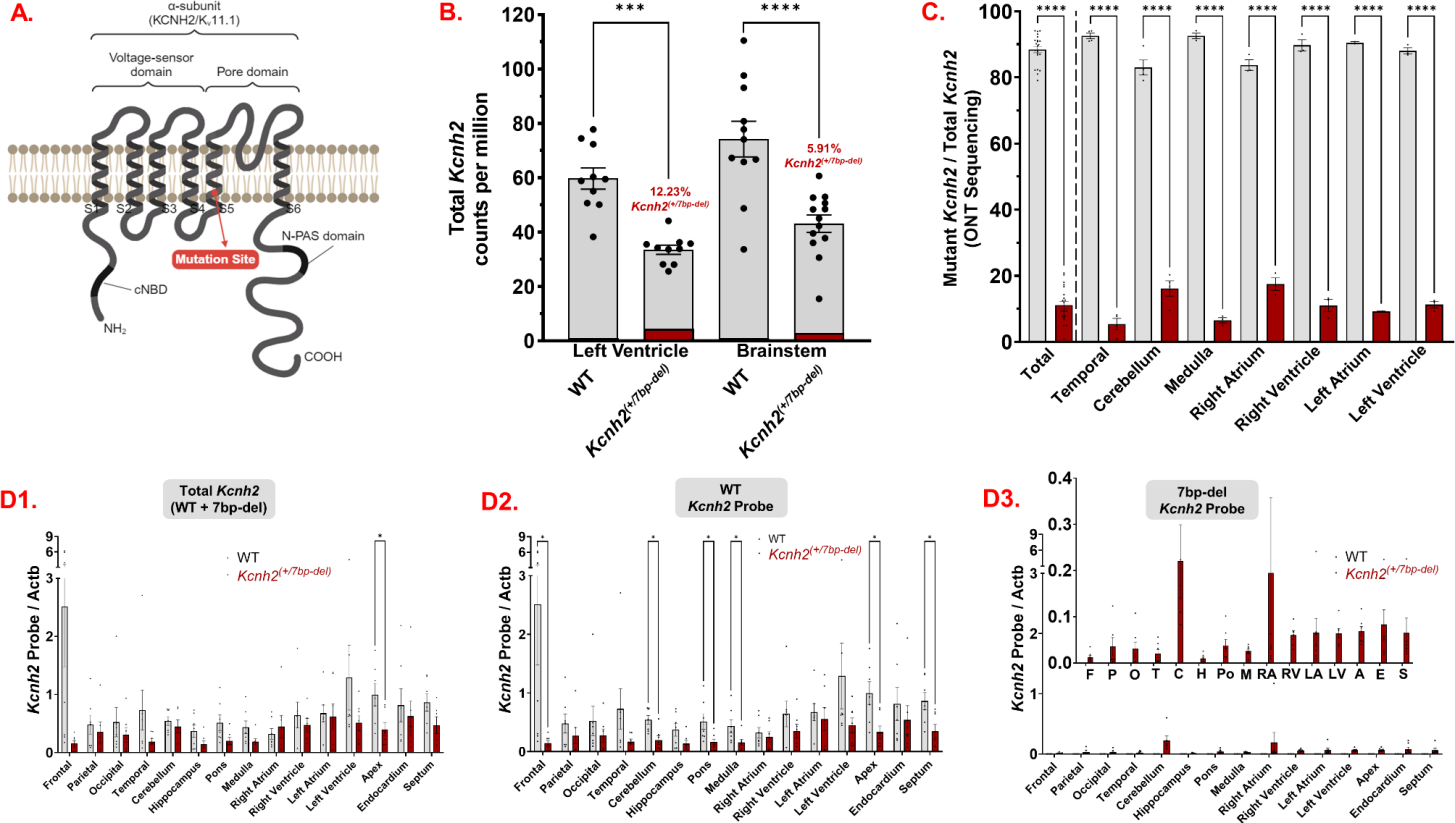
CRISPR Cas-9-mediated *Kcnh2* mutation leads to altered *Kcnh2* expression patterns. (**A).** Topology of K_v_11.1 protein denoting the site of 7bp-deletion mutation in S5 of the pore domain. (**B).** RNA sequencing indicates altered total *Kcnh2* expression in WT and *Kcnh2*^(+/7bp−del)^ rabbit heart and brain tissue. WT left ventricle *N* = 10 rabbits, WT brainstem *N* = 11 rabbits, *Kcnh2*^(+/7bp−del)^ left ventricle *N* = 10 rabbits, *Kcnh2*^(+/7bp−del)^ brainstem *N* = 13 rabbits. (**C).** Oxford Nanopore Technology (ONT) sequencing of WT and mutant *Kcnh2* amplicons generated using RNA extracted from mutant rabbits (*N* = 3). (**D).** qPCR resuls for region-specific expression of **(1)** Total (WT + mutant), **(2)** WT, and **(3)** 7bp-del mutant *Kcnh2* transcripts in WT (*N* = 7) vs. mutant (*N* = 7) rabbit tissue. All qPCR data is normalized to plate normalization factor (*described in results*). Statistical analyses: performed one-way ANOVA (B, C) and unpaired t-tests (D1, 2). *, *p* < 0.05; **, *p* < 0.01; ****, *p* < 0.0001. Actb: beta-actin

*CRISPR-Generated Knock-In LQT2 Rabbit Model*: *Kcnh2*^(+/7bp−del)^ rabbits were generated with a 7bp deletion in the S5 pore domain of the rabbit endogenous *Kcnh2* gene (Fig. 1A), which creates 17 predicted premature stop codons. Mutant gDNA is present in tissue collected from the offspring of *Kcnh2*^(+/7bp−del)^, but not WT rabbits (Supplementary Fig. 2A). The mutation is germline and the *Kcnh2*^(+/7bp−del)^ offspring are viable and fertile. Despite multiple rounds of breeding 2 *Kcnh2*^(+/7bp−del)^ rabbits together, there were only *Kcnh2*^(+/7bp−del)^ and WT offspring; there were no *Kcnh2*^(7bp−del/7bp−del)^ rabbits.

### WT/Mutant Kcnh2 expression patterns:

RNA sequencing results indicate a 43.9% reduction of total *Kcnh2* in the heart (left ventricle) and 41.9% reduction in the brain (brainstem) in *Kcnh2*^(+/7bp−del)^ vs. WT tissue (Fig. 1B). In *Kcnh2*^(+/7bp−del)^ rabbits, mutant transcript comprises 12.2% and 5.9% of total *Kcnh2* transcript in the heart and brain, respectively. Similarly, ONT sequencing results demonstrate that mutant transcript is 11% of the total *Kcnh2* (Fig. 1C, range 5.4–16.1% in specific brain & heart regions).

We profiled tissue-specific total, WT, and mutant *Kcnh2* expression using qPCR. The primers amplify the mutant region in both WT and mutant tissue, and the primers/probes are specific to WT and mutant cDNA (Supplementary Figs. 2 & 3). Results from qPCR indicate that total and WT *Kcnh2* is lower in each region of the brain and heart of *Kcnh2*^(+/7bp−del)^ vs. WT rabbits (Fig. 1D1-2). In *Kcnh2*^(+/7bp−del)^ rabbits, total *Kcnh2* is significantly lower in the heart apex, and WT *Kcnh2* transcript is significantly lower in the frontal cortex, cerebellum, pons, and medulla of the brain, as well as the heart apex and septum. Mutant *Kcnh2* is detected in each of the brain and heart regions, with the highest expression in the cerebellum and right atrium (Fig. 1D3). In summary, results from RNA sequencing, ONT sequencing, and qPCR demonstrate that total and WT *Kcnh2* expression are each significantly lower in *Kcnh2*^(+/7bp−del)^, compared to WT tissue. Mutant *Kcnh2* comprises a small percentage of the total *Kcnh2* in *Kcnh2*^(+/7bp−del)^ tissue. Transcriptome wide RNA sequencing results are included in Supplementary Fig. 4. 

*K*_*v*_*11.1 Protein Expression*: We raised an antibody in guinea pigs against a peptide corresponding to the C-terminus of K_v_11.1 (Supplementary Table 1). The antibody is immunoreactive against full-length mammalian K_v_11.1^WT^. It recognizes N-terminally tagged exogenous rabbit MYC-K_v_11.1^WT^ at ~ 145 kDa, but not FLAG-Kv11.1^7bp−del^ in HEK cells (Supplementary Fig. 5A). Anti-MYC yields only a band at ~ 145 kDa in MYC-Kv11.1^WT^. Consistent with the 7bp frameshift deletion creating predicted premature stop codons, a ~ 72 kDa band is detected in the FLAG-K_v_11.1^7bp−del^ samples using anti-FLAG, but not anti-K_v_11.1 (C-terminal epitope). This K_v_11.1 antibody also detects endogenous K_v_11.1^WT^ in mouse pituitary and human neuroblastoma cell lines (α-T3 & SH-SY5Y, Supplementary Fig. 5B).

**Fig. 2:**
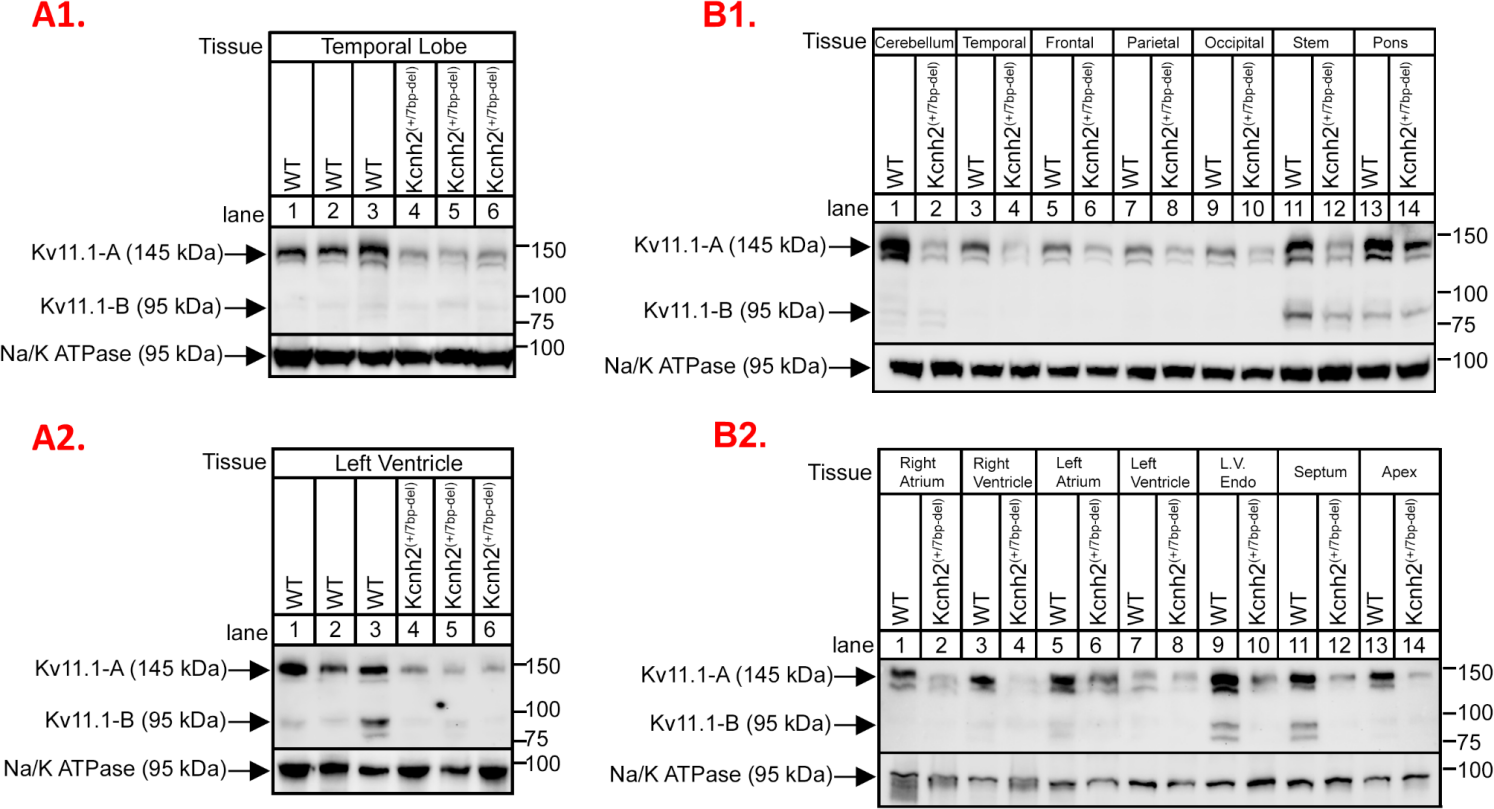
K_v_11.1 expression in rabbit tissue. **(A).** Immunoblot showing immunoreactivity of C-terminus-specific K_v_11.1 antibody recognizing full-length K_v_11.1^WT^ in WT (*N* = 3) and *Kcnh2*^(+/7bp−del)^ (*N* = 3) **(1)** heart (left ventricle) and **(2)** brain (temporal lobe) tissue. (**B).** Immunoblot showing region-specific expression of full-length K_v_11.1^WT^ in WT and *Kcnh2*^(+/7bp−del)^ **(1)** heart and **(2)** brain tissue

Membrane preparations from the heart and brain of WT and *Kcnh2*^(+/7bp−del)^ rabbits, indicate immunoreactive bands at 145-150 kDa (Fig. 2A). K_v_11.1 expression is markedly lower in the temporal lobe (Fig. 2A1) and left ventricle (Fig. 2A2) of *Kcnh2*^(+/7bp−del)^ vs. WT rabbit samples (*n* = 3 rabbits/genotype). Interestingly, K_v_11.1 expression levels vary in specific regions of the WT brain (highest in cerebellum, brainstem and pons), but is consistently lower in *Kcnh2*^(+/7bp−del)^ tissue (Fig. 2B1). K_v_11.1 expression is consistenty lower in each of the regions of the heart of *Kcnh2*^(+/7bp−del)^ vs. WT rabbits (Fig. 2B2). Overall, full-length K_v_11.1 expression is lower throughout the brain and heart of *Kcnh2*^(+/7bp−del)^ vs. WT rabbits.

### In vivo characteriztion of cardiac electrical function:

**Fig. 3:**
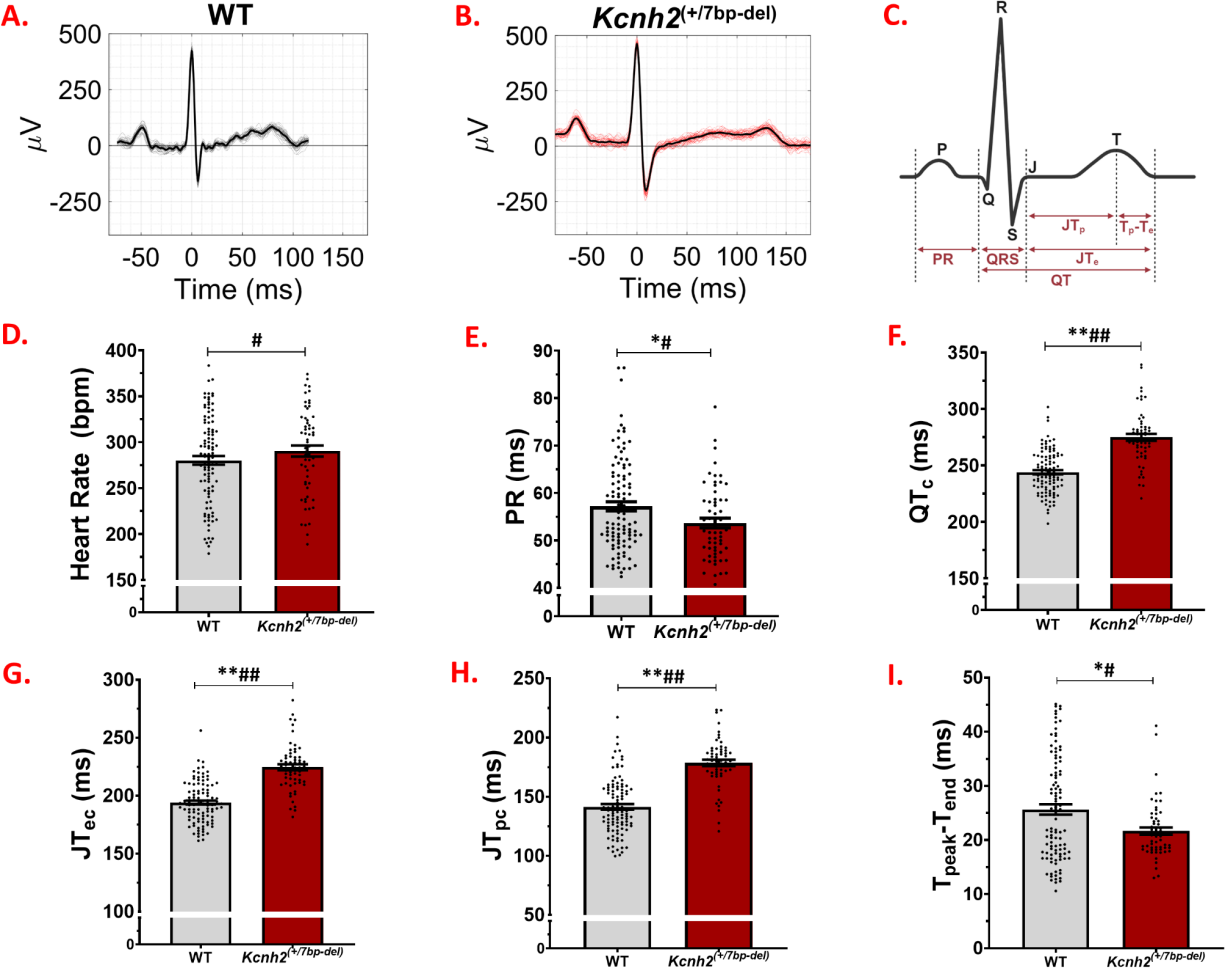
In-vivo cardiac phenotype in LQT2 knock-in rabbits. Representative ECG traces of (**A).** WT (black) and (**B).***Kcnh2*^(+/7bp−del)^ (red) rabbits, showing the P, QRS, and T waves. (**C).** Schematic representing ECG intervals that are quantified. Depolarization and repolarization metrics in WT (grey, *N* = 68 rabbits, *n* = 89 recordings) and *Kcnh2*^(+/7bp−del)^ (red, *N* = 37 rabbits, *n* = 52 recordings) rabbits: (**D).** Heart rate, (**E).** PR, **(F).** QT_c_, (**G).** JT_ec_, (**H).** T_pc_, and (**I).** T_peak_-T_end_. Statistical analyses: *, *p* < 0.05; ** *p* < 0.0001 Wilcoxon rank-sum test. ^#^, *p* < 0.05; ^##^, *p* < 0.0001 logistic regression models adjusted for age, sex, and heart rate

Baseline conscious restrained video/EEG/ECG recordings were performed in WT and *Kcnh2*^(+/7bp−del)^ rabbits. Figure 3A and B are 40-beat average ECG waveforms from WT and *Kcnh2*^(+/7bp−del)^ rabbits. In contrast to rodents [14], it resembles the human ECG waveform, as seen by positive and broad T-waves. The widely-split and notched (bifid), and prolonged T-wave morphology in the *Kcnh2*^(+/7bp−del)^ ECG trace reproduces the T-wave morphology seen in people with LQT2 [15–17].

We assessed heart rate, conduction, and repolarization metrics in WT vs. *Kcnh2*^(+/7bp−del)^ rabbits (Fig. 3C-I, Supplementary Table 2). P and QRS durations are similar in WT vs. *Kcnh2*^(+/7bp−del)^ rabbits. Atrio-ventricular conduction time (PR interval) is shorter in *Kcnh2*^(+/7bp−del)^ vs. WT Rabbits (*p* = 0.018, Fig. 3E). Heart rate corrected repolarization measures, which include QT_c_ (*p* < 0.001, Fig. 3F), JT_ec_ (*p* < 0.001, Fig. 3G), and JT_pc_ (*p* < 0.001, Fig. 3H), are longer in *Kcnh2*^(+/7bp−del)^ (*N* = 37 rabbits, *n* = 52 recordings), vs. WT rabbits (*N* = 68 rabbits, *n* = 89 recordings). The T_peak_-T_end_ is shorter in *Kcnh2*^(+/7bp−del)^ vs. WT rabbits (*p* < 0.05, Fig. 3I), which indicates reduced spatial and transmural dispersion of repolarization [18, 19]. Similar results were also seen when stratifying for age (Supplementary Fig. 6). Logistic regression models adjusting for age, sex, and heart rate further confirm that PR, QT_c_, JT_ec_, and JT_pc_ are prolonged, and T_peak_-T_end_ is shorter in *Kcnh2*^(+/7bp−del)^ vs. WT rabbits. In summary, *Kcnh2*^(+/7bp−del)^ rabbits reproduce the human LQT2 ECG pathology of significant prolongation of cardiac ventricular repolarization metrics(QT_c_, JT_pc_, & JT_ec_).

### In vivo characterization of EEG recordings:

**Fig. 4:**
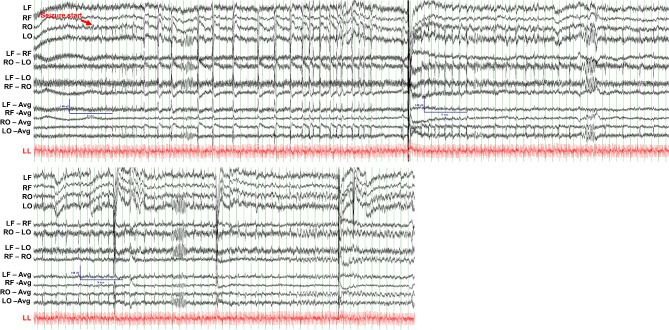
Representative EEG (black) and ECG (red) traces during an electrographic seizure in a 2-week-old male *Kcnh2*^(+/7bp−del)^ rabbit; epileptiform discharges in right occipital region increasing in amplitude over time. Red arrow indicates seizure start. LF: left frontal, RF: right frontal, RO: right occipital, LO: left occipital, LL: left leg, RA: right arm, LA: left arm. Scale bar shown for EEG signal: 140µV amplitude, 6 s

**Fig. 5:**
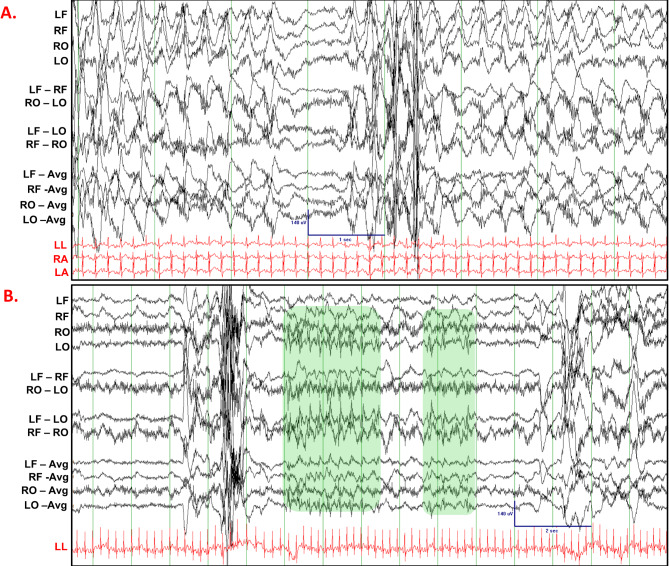
In-vivo neuronal phenotype in WT and *Kcnh2*^(+/7bp−del)^ rabbits. (**A).** Spontaneous epileptiform discharges and a clonic seizure noted in a 7-week-old female *Kcnh2*^(+/7bp−del)^ rabbit. EEG scale bar indicates 140µV and 1 s. (**B).** EEG-ECG traces between myoclonic jerks showing epileptiform discharges with a spike and slow wave morphology. EEG scale bar indicates 140µV and 2 s

**Fig. 6:**
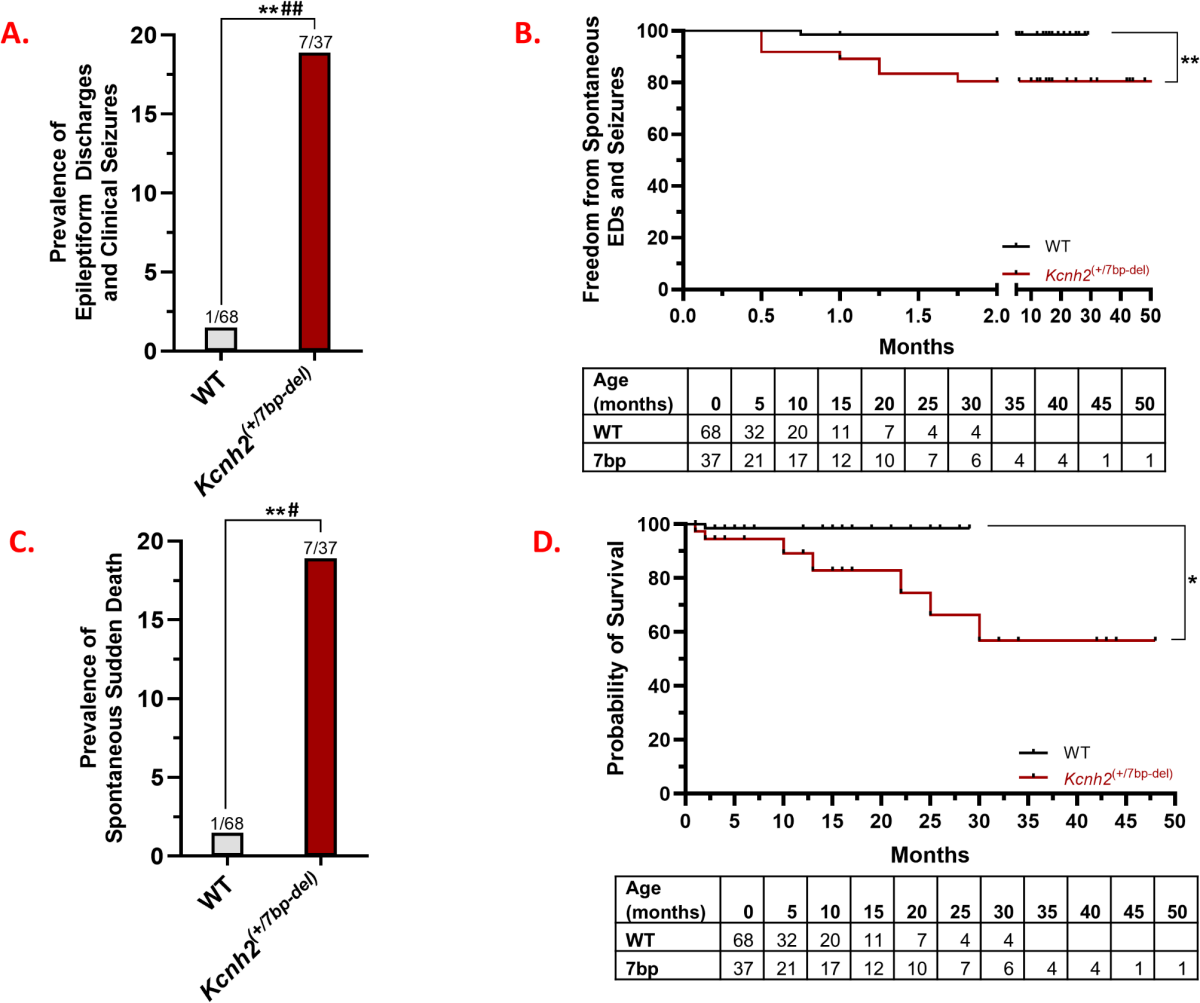
(**A).** Prevalence of epileptiform discharges and clinical seizures (WT *N* = 68, *n* = 140; *Kcnh2*^(+/7bp−del)^*N* = 37, *n* = 85). (**B).** Kaplan-Meier curves of in vivo spontaneous neuronal phenotype over the lifetime of WT and *Kcnh2*^(+/7bp−del)^ rabbits. (**C).** Prevalence of spontaneous sudden death in WT (*N* = 68) and *Kcnh2*^(+/7bp−del)^ (*N* = 37) rabbits. (**D).** Kaplan-Meier survival curves of spontaneous sudden death events over the natural-lab lifetime of WT and *Kcnh2*^(+/7bp−del)^ rabbits. Statistical analyses: performed Fisher’s exact test (**A**, **C**) and Mantel-Cox log-rank test (**B**, **D**). *, *p* < 0.05; **, *p* < 0.01. ^#^, *p* < 0.05; ^##^, *p* < 0.01 logistic regression models adjusted for age and sex. N = number of animals, n = number of recordings. LF: left frontal, RF: right frontal, RO: right occipital, LO: left occipital, LL: left leg, RA: right arm, LA: left arm

**Fig. 7:**
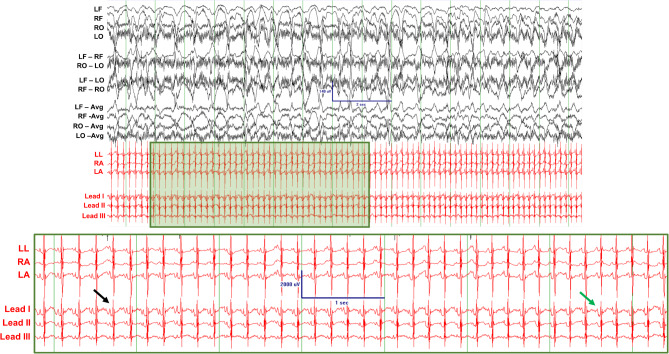
Sudden Death Case-1 exhibiting epileptiform discharges and extreme QT prolongation. Black arrow indicates a region where T and P waves of adjacent beats are temporally separated. Green arrow indicates a region where, due to extreme QT prolongation, the P wave is encapsulated by the T wave of a previous beat, causing an absence of the T-P interval. EEG scale bar indicates 140µV amplitude and 2 s; inset: ECG scale bar indicates 2000µV amplitude and 1 s

EEG recordings were manually reviewed to identify epileptiform activity and seizures (*N* = 105 rabbits, *n* = 225 30–60 min recordings). We followed the criteria for inter-ictal epileptiform discharges set by the American Academy of Neurology [20]. Supplementary Fig. 1 shows a normal EEG-ECG recording from a 1.5-month-old male WT rabbit. We identified several instances of spontaneous epileptiform activity in *Kcnh2*^(+/7bp−del)^ rabbits. Interestingly, all of the cases of epileptiform activity and seizures are noted in juvenile rabbits (2–8 weeks of age). Figure 4 shows an EEG recording from a 2-week-old male *Kcnh2*^(+/7bp−del)^ rabbit; it illustrates spontaneous epileptiform activity and an electrographic seizure. The red arrow indicates the start of the seizure, followed by the temporal evolution of epileptiform discharges that originate in the right occipital region, increase in amplitude, and are then seen in the left occipital leads. In a 7-week-old female *Kcnh2*^(+/7bp−del)^ rabbit, spontaneous epileptiform discharges and clonic head movement is noted (Fig. 5A). In a 4-week-old female *Kcnh2*^(+/7bp−del)^ rabbit, spike and slow wave epileptiform activity is noted in between myoclonic head jerks (Fig. 5B). In summary, spontaneous epileptiform activity and seizures are identified in 7 of 37 *Kcnh2*^(+/7bp−del)^ vs. 1 of 68 WT rabbits (Fig. 6A; *p* < 0.01). Kaplan Meier analysis and stratifcation for age indicates that all cases occur in juvenile rabbits (< 3 months of age), resulting in a significantly reduced freedom from epileptiform discharges and motor seizures in *Kcnh2*^(+/7bp−del)^ rabbits (Fig. 6B; WT vs. *Kcnh2*^(+/7bp−del)^ log-rank test, *p* < 0.01, Supplemenentary Fig. 7A). Logistic regression models adjusting for age and sex further confirm that *Kcnh2*^(+/7bp−del)^ rabbits demonstrate an increased risk of spontaneous epileptiform discharges and seizures, compared to WT rabbits.

*Increased Prevalence of Spontaneous Sudden Death in Kcnh2*^(+/7bp−del)^*Rabbits*: There is a 13-fold higher prevalence of spontaneous sudden death in *Kcnh2*^(+/7bp−del)^ (7 of 37 rabbits), compared to WT rabbits (1 of 68 rabbits, Fig. 6C; *p* < 0.01). Kaplan Meier analysis and stratification for age indicates that spontaneous sudden death events are observed at both juvenile and adult timepoints (Fig. 6D; WT vs. *Kcnh2*^(+/7bp−del)^ log-rank Test, *p* < 0.05, Supplementary Fig. 7B). Logistic regression models adjusting for age and sex further confirm that *Kcnh2*^(+/7bp−del)^ rabbits demonstrate an increased risk of spontaneous sudden death, compared to WT rabbits. During necropsy, all of the rabbits were in rigor, despite 5 of the deaths either being witnessed or the rabbit was last seen alive ≤ 2 h prior. We did not identify an apparent cause of death during external examination in the cage or during the necropsy. We ruled out structural heart disease, trauma or internal bleeding, overt infection, tumor, and gastrointestinal blockage. There was no sudden large weight change noted prior to death. Death occurred between the ages of 0.4 to 30 months of age. Sudden death was witnessed/videoed for 3 *Kcnh2*^(+/7bp−del)^ (Sudden Death Cases 1–3) and 1 WT rabbit (Sudden Death Case 8), and there are video/EEG/ECG recordings for Sudden Death Cases 1 and 2 (Table 1).

Sudden Death Case 1 succumbed to status-epilepticus-mediated sudden death. We examined the prevalence of epileptiform activity. Recordings acquired 24 and 48 h prior to death indicate the presence of epileptiform activity. 2 of 3 mutant littermates also exhibited epileptiform activity, while no EEG abnormalities were noted in the 2 WT littermates. In this litter, the QT_c_ is longer in the 4 mutant vs. 2 WT rabbits (*p* < 0.0001, Supplementary Table 3A). Interestingly, the inter-ictal QT_c_ for Sudden Death Case 1 is longer on the day of sudden death, compared to inter-ictal recordings 24 and 48 h prior to sudden death (*p* < 0.0001, Supplementary Table 3B). During a seizure, the QT_c_ prolongation was so extreme that the P-wave is often hidden in the T wave of the previous beat (Fig. 7).

**Fig. 8:**
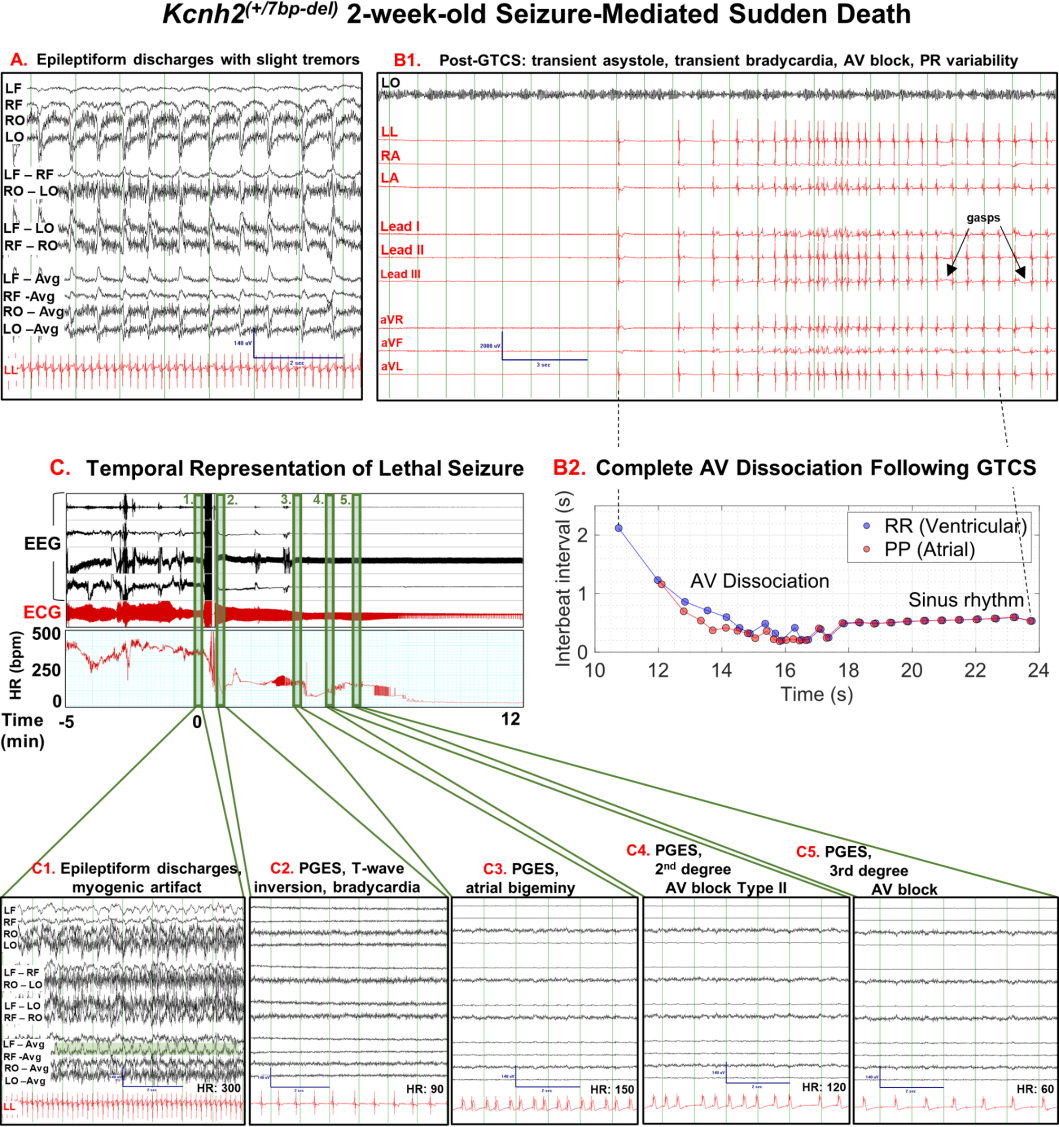
Sudden Death Case-1: Status-epilepticus-mediated sudden death in a 2-week-old female *Kcnh2*^(+/7bp−del)^ rabbit. (**A).** Representative EEG (black) and ECG (red) traces showing epileptiform discharges and tremors ~ 1.5 h prior to sudden death. Scale bar shown for EEG signal: 140µV amplitude, 2 s. (**B1).** Cardiac manifestations following a GTCS using one EEG trace, 3 referential ECG traces, and the standard bipolar and augmented ECG lead configurations (I, II, III, aVR, aVL, & aVF). Immediately following GTCS: sinus pause (~ 8 s), transient bradycardia, AV block, and inverted T-waves. Rabbit was apneic for ~ 19 s post-GTCS until first gasp (marked by arrow). Scale bar shown for ECG signal; 2000µV amplitude, 3 s. (**B2).** Progression of cardiac abnormalities and recovery following a convulsive seizure. Ventricular rhythm with each QRS complex and atrial rhythm with each P wave plotted over time. (**C).** Lethal seizure is depicted at minute zero. A cardiac tachogram is shown starting at 5 min pre-lethal seizure and ending close to asystole at 12 min post-lethal seizure. **(1)** Pre-ictal interval shows epileptiform activity (highlighted in green), myogenic artifact from tremors, and heart rate at ~ 300 BPM. Post-ictal intervals show **(2)** PGES, bradycardia (HR: ~90 BPM) and inverted T-waves, **(3)** PGES and atrial bigeminy, **(4)** PGES and 2nd degree AV block, and **(5)** PGES and 3rd degree AV block. Scale bars shown for EEG signal; 140µV amplitude, 2 s

On the day of sudden death, there are numerous instances of epileptiform activity, electrographic seizures, and epileptic seizures with profound clinical manifestations. The rabbit developed seizure clusters that included focal myoclonic seizures that transitioned to generalized tonic-clonic seizures (GTCS), which included periods of tonic limb extension and rhythmic movement of all extremities. Figure 8A shows rhythmic ~ 1.5 Hz epileptiform discharges during a period that the rabbit presented with slight tremors. Amidst a background of myogenic artifact, there are EEG spikes that are higher in amplitude than the background, and the spikes have fast upstrokes followed by slower downstrokes. Following a particularly severe GTCS, there was a period of transient asystole lasting approximately 10 s (Fig. 8B1). Transient asystole was followed by consecutive ventricular escape beats, then transient bradycardia and approximately 5 s of complete heart block (atrio-ventricular, AV, dissociation, Fig. 8B2, RR and PP intervals plotted over time). As sinus rhythm returned, there was AV conduction (PR interval) variability in the first 7 beats, followed by stable atrial and ventricular rates and consistent PR intervals (Supplementary Fig. 8A-D). Exertional gasp movements of the chest were visible and detected on the ECG (Fig. 8B1). Video 1 illustrates the lethal event, which includes the pre-ictal period with epileptiform discharges, the onset of a GTCS, and the EEG/ECG manifestations during the post-ictal period and leading up to asystole/death. Following the lethal GTCS, Sudden Death Case 1 displayed post-ictal generalized EEG suppression (PGES, Fig. 8C2-5), apnea (based on video), inverted T-waves on the ECG (indicative of transient ischemia, Fig. 8C2), bradycardia (Fig. 8C2-5), atrial bigeminy (couplets of beats, Fig. 8C3), 2nd degree AV block Type-2 (each QRS is preceded by a P-wave but QRS randomly drop, Fig. 8C4), 3rd degree AV block (AV dissociation, Fig. 8C5) with focal ventricular activity, and terminal asystole. The rabbit went into rigor within 5 min following the lethal seizure.

Sudden Death Case 2 is a 1.8-month-old *Kcnh2*^(+/7bp−del)^ rabbit that suffered sudden cardiac death while collecting a routine 4 mm ear biopsy for genotyping. Sudden stress is a known trigger for arrhythmias in people with LQT2 [21]. Several cardiac pathologies were noted, which included atrial bigeminy, frequent periods of bradycardia (< 96 bpm), ECG ST-elevation/depression (indicative of ischemia, Supplementary Fig. 9A & 10), premature ventricular complexes (PVCs), 2nd degree AV block, and ultimately asystole. The rabbit also developed cardiogenic seizures. The clinical manifestations included myoclonic activity, however no epileptiform discharges were noted surrounding the convulsive seizures.

Sudden Death Case 3 is an 11-month-old female *Kcnh2*^(+/7bp−del)^ rabbit that died suddenly during a routine health check blood draw, likely also due to a stress-mediated response. The rabbit convulsed vigorously and went into rigor within 5 min of the lethal event (Table 1). Since there were not video-EEG-ECG recordings during this event, we cannot confirm whether the clinical manifestations were due to an epileptic or cardiogenic seizure.

Sudden Death Case 5 is a 21.7-month-old female *Kcnh2*^(+/7bp−del)^ rabbit that was found dead in the housing cage, 2 h after last seen alive. The rabbit was found in full rigor and had a history of ECG abnormalities including a high incidence of PVCs (0.68/min, Supplementary Fig. 9B). No EEG abnormalities were noted in several recordings throughout the rabbit’s life.

Sudden Death Case 8 is a 2-month-old female WT rabbit that suffered a convulsive episode leading to death in the housing cage during the night. Continuous audio-video recording indicates episodes of tonic head extension and non-rhythmic dysynchronous forelimb motor manifestations prior to death, which is consistent with convulsive syncope. The rabbit was found deceased ~ 7 h post-mortem in full rigor. During a video/EEG/ECG recording ~ 5 h prior to sudden death, the rabbit exhibited PVCs (0.59/min), sinus pause (> 2-sec), ST elevation, and deep inverted T-waves. In a recording taken 1 month prior to sudden death event, the rabbit exhibited several interictal epileptiform discharges.

In all other cases of spontaneous sudden death, the rabbits were found deceased unexpectedly in their housing cage and in full rigor (Table 1). In summary, there is a significantly higher prevalance of sudden death in *Kcnh2*^(+/7bp−del)^ vs. WT rabbits. While the cause of death was not definitive in all cases, none of the rabbits exhibited any findings during necropsy that would rule out SUDEP (e.g., structural abnormalities). In 3 cases of sudden death, we captured seizures and/or ECG abnormalities, which indicates the role of neuro-cardiac electrical abnormalities leading to spontaneous sudden death.

**Table 1:** Detailed description of all cases of spontaneous sudden death. *: all animals that died suddenly and spontaneously were found in rigor, some within 2 h of last being seen alive. Post-mortem necropsy did not reveal any anatomical or toxicological cause of death in any case of sudden death. SCD: sudden cardiac death

Case #	Genotype	Age (months)	Sex	Time from last seen alive	Rigor*	Necropsy Findings	History of Epileptiform Activity	Manifestations at Time of Death	Mechanism of Sudden Death
1	*Kcnh2* ^(+/7bp−del)^	0.4	Female	Witnessed & Recorded	Yes	None	Yes	Myoclonic jerks, GTCS, tonic limb/head extension, PGES, bradycardia, ECG T-wave inversion, 2nd & 3rd degree AV block, cardiac focal activity,& asystole	Status Epilepticus
2	*Kcnh2* ^(+/7bp−del)^	1.8	Male	Witnessed & Recorded	Yes	None	No	Atrial bigeminy, bradycardia, ECG ST-elevation/depression (ischemia), PVCs, 2nd degree AV block, asystole	SCD
3	*Kcnh2* ^(+/7bp−del)^	11	Female	Witnessed	Yes	None	N/A	Loss of consciousness with convulsions during routine blood draw	unknown
4	*Kcnh2* ^(+/7bp−del)^	13.4	Female	< 24 h	Yes	None	No	Found in the cage in rigor	unknown
5	*Kcnh2* ^(+/7bp−del)^	21.7	Female	2 h	Yes	None	No	Found in the cage in rigor	unknown
6	*Kcnh2* ^(+/7bp−del)^	25.3	Female	< 7 h	Yes	None	N/A	Found in the cage in rigor	unknown
7	*Kcnh2* ^(+/7bp−del)^	30	Male	< 2 h	Yes	None	No	Found in the cage in rigor	unknown
8	*Kcnh2* ^(+/+)^	2	Female	Video Recorded	Yes	None	Yes	Convulsive Syncope	SCD

## Discussion

We developed the first translational knock-in model of *Kcnh2*-mediated LQT2. This study provides a comprehensive characterization of the molecular, biochemical, and in vivo neuro-cardiac phenotypes in *Kcnh2*^(+/7bp−del)^ rabbits, which mirror the cardiac and neuronal pathologies, and sudden death (i.e., SCD & seizure-mediated) seen in people with LQT2. There is a ~ 50% reduction in total and WT *Kcnh2*, and full-length WT K_v_11.1 in *Kcnh2*^(+/7bp−del)^ rabbits. Consistent with LOF *Kcnh2* variants [22, 23], there is a significant prolongation of ventricular repolarization in *Kcnh2*^(+/7bp−del)^ vs. WT rabbits. *Kcnh2*^(+/7bp−del)^ rabbits show an increased prevalence of spontaneous epileptiform discharges, electrographic seizures, and motor seizures, including myoclonic activity, clonic seizures, and GTCS. *Kcnh2*^(+/7bp−del)^ rabbits show an increased prevalence of spontaneous sudden death. These events include cases of SCD preceded by convulsive syncope, and seizure-mediated sudden death preceded by PGES and ECG abnormalities.

People with LQT2 are at an increased risk of both cardiac arrhythmias and seizures [1, 9]. Interestingly, 13% of SUDEP cases have variants associated with LQTS, including *KCNH2* [24]. This translational model facilitates comprehensive assessments of the implications of *Kcnh2* variants in all organ systems, particularly the brain.

*Kcnh2*^(+/7bp−del)^ rabbits are more translationally relevant than cellular or rodent models, and facilitate studies that are not feasible in people. Heterologous cellular models expressing ion channel proteins of interest lack the native cellular machinery, accessory proteins, and physiological environment of the host [14]. Expression is often at non-physiological levels, which generate results that are inconsistent with people [14]. Human induced pluripotent stem cell-derived cardiomyocytes (hiPSC-CMs) are not fully mature, exhibit spontaneous activity, have a depolarized resting membrane potential, slow AP upstroke velocity, and the AP morphology more closely resembling fetal, rather than adult human cardiomyocytes [25]. Cardiac ion channel expression, AP morphology, heart rate, and ECG parameters are very different in rodents vs. humans [14]. Thus, the utility of mice as a model of cardiac arrhythmias and SCD remains controversial [26]. While cardiac repolarization is driven by I_Kr_ in humans and rabbits, the transient outward and ultra-rapid potassium currents (I_to_ & I_Kur_) are the major repolarizing currents in rodents [27–29].

Several genetic mouse models of LQT2 have been generated with varying phenotypes [30–33]. (1) Adult mice that overexpress the *Kcnh2-*G628S exhibit complete loss of I_Kr_. Yet, the mutant mice do not exhibit any changes in the AP duration, ECG intervals (e.g., QT_c_), or the susceptibility to pacing-induced arrhythmias [30]. (2) Homozygous ERG1-B (*Kcnh2* isform B) knockout mice have no I_Kr_, but there is no difference in any ECG metrics in neonatal and adult mutant vs. WT mice [31].(3) Mice with homozygous deletion of the K_v_11.1 S4-S6 domain are embryonically lethal with abnormalities in embryogenesis. Neonatal, but not adult, heterozygous mutant mice exhibit QT_c_ prolongation. (4) Mice homozygous for *Kcnh2*-N629D are embryonically lethal due to defects in cardiogenesis and vasculogenesis [32, 33]. There is complete loss of I_Kr_ and prolonged AP durations in homozygous mutant myocytes. In contrast, 76.3% of myocytes from heterozygous mutant embryos exhibit WT-like I_Kr_ and AP morphology [32]. In contrast, juvenile and adult *Kcnh2*^(+/7bp−del)^ rabbits exhibit QT_c_ prolongation and SCD, and thus are a translational model of LQT2.

Several rodent models of epilepsy do not model the natural progression of seizure onset and are not physiologically relevant; these models require triggers to induce seizures [34, 35], have conditional, organ, or cell-type specific knock-out of genes of interest [36], do not reproduce the natural progression of clinical epilepsy, or have a high mortality rate that is not comparable to people with the type of epilepsy being modeled [37]. Similar to our previous study that showed that 18% of people with LQT2 have a history of seizures/epilepsy, 19% of *Kcnh2*^(+/7bp−del)^ rabbits develop spontaneous epileptiform activity and seizures [9].

Rabbits provide a valuable model of cardiac arrhythmias and epilepsy, and are ideal for drug testing [29]. Cardiac repolarization is driven by I_Kr_ in both humans and rabbits [29]. The cardiac electrical-activation recovery process is similar, as illustrated by similar AP and ECG morphologies, and sensitivity to I_Kr_ blockade [29]. Thus, the rabbit heart provides an excellent translational tool for drug development and cardiac safety testing, particularly for evaluating if a drug has off-target effects on K_v_11.1 function, and leads to QT_c_ prolongation, arrhythmias, and sudden cardiac death [38]. As such, several approaches have been designed to study the cardiac electrophysiological effects of drugs using rabbits, such as the in vitro Purkinje fiber assay to study conduction block potential [39], ex vivo Langendorff-perfusion to investigate arrythmia mechanisms [40, 41], and in vivo transgenic models to represent the human heart with all its innervations intact [41].

A cardiac-specific transgenic rabbit model of LQT2 exhibits prolonged QT and AP durations, reduced I_Kr_, polymorphic ventricular tachycardia, and high rate of SCD [41]. However, these mutant rabbits have cardiac-specific overexpression of mutant human *Kcnh2*, in addition to the endogenous rabbit WT *Kcnh2* [41]. This is in contrast to humans that only have 2 copies of each allele, *Kcnh2* expression is not driven by the beta-myosin heavy chain promoter and overexpressed in specific organs, and the genetic variant is present wherever the gene/protein is normally expressed. Importantly, the *Kcnh2*^(+/bp−del)^ rabbits overcome limitations posed by cellular, rodent, or cardiac-specific transgenic rabbit models of LQT2. The CRISPR-Cas9-mediated knock-in 7bp frameshift deletion is in one allele of the endogenous rabbit *Kcnh2*, which better models the genetics seen in people with LQT2, and alters *Kcnh2*/K_v_11.1 expression and electrical function wherever *Kcnh2* is naturally expressed.

In the heart, loss-of-function variants in *KCNH2* cause cardiac AP and ECG-QT_c_ prolongation through a reduction in I_Kr_ density and/or alteration of the channel’s biophysical properties [42]. The reduction in the repolarization reserve causes an imbalance between the cardiac depolarizing and repolarizing forces, leading to an increased risk of polymorphic ventricular tachycardia, ventricullar fibrillation, and sudden cardiac death [43]. In the brain, pharmacological reduction in I_Kr_ depolarizes the resting membrane potential [44], reduces AP interspike intervals [45], and reduces spike-frequency adaption in neurons [45]. Ultimately, this causes neuronal burst firing and hyperexcitability, which underlies epileptogenesis [46].

All rabbits that died suddenly were found in rigor (between 2 and 24 h of last being seen alive). Rigor is due to ATP depletion, which can occur due to pre-mortem convulsive activity. We observed that in cases of pro-convulsant drug-induced seizure-mediated sudden death, there is a rapid onset of rigor in < 30 min. This is in contrast to rabbits that are euthanized and do not go into rigor for > 2 h post-mortem. Rigor reaches 50% of maximum at > 3.5-hours at 37ºC, > 6.5-hours at room temperature (17ºC), and peak rigor is at > 8-hours and > 10-hours, respectively [47]. Thus, many of the unwitnessed cases of sudden death were likley preceeded by vigorous muscle activity-mediated sudden depletion of ATP.

The mechanism underlying SUDEP is very complex and involves a temporal cascade of multisystem dysfunction [48]. In a cohort of SUDEP cases that occured in epilepsy monitoring units, all deaths occurred following a generalized tonic-clonic seizure, which was accompanied by neuronal, cardiac, and respiratory dysfunction [49]. Following the seizure, 90% of SUDEP cases demonstrated cardiorespiratory dysfunction, characterized by bradycardia, transient (> 5 s) or terminal apnea, and transient (> 10 s) or terminal asystole. Cardiac arrhythmias are a proposed mechanism of SUDEP [12]. In patients with epilepsy, including those that later suffered SUDEP [50], peri-ictal arrhythmias were recorded, including atrial fibrillation, asytole, supraventricular tachycardia, and bundle branch block [50, 51]. Seizure duration was longer in patients with vs. without simultaneous cardiac abnormalities [51]. Both long seizure duration, such as status epilepticus, and multi-system pathologies increase the risk of sudden death [49, 52]. Ion channelopathies that increase the risk of both epilepsy and cardiac disease are found in SUDEP cases [24, 53]. Specifically, when compared to an epilepsy control population of 50 years or older (i.e. those who are thought to have “escaped” SUDEP), the prevalence of LOF *KCNH2* variants was significantly higher in the SUDEP cohort [54]. Another recent study reported that an optimal cut-off QT_c_ interval predicts mortality in people with epilepsy [55]. Variants in genes linked to Brugada syndrome (*SCN5A*), LQT3 (*SCN5A*), and Dravet syndrome (*SCN1A*) are also reported in SUDEP cohorts [12]. *KCNH2*-LOF-mediated LQT2, epilepsy, and sudden death was reported in a family with a heterozygous point mutation (c.246T > C, p.I82T) [56]. The SUDEP case and twin sister showed generalized spike and slow wave EEG complexes, QT prolongation (550ms), and abnormal T-wave morphology. The *KCNH2* variant caused a significant decrease in I_Kr_ density and faster channel deactivation kinetics, confirming severe LOF [56]. People with LQT2 exhibit theta activity, focal and bilateral discharges, and epileptiform discharges arising from the left/right regions and bilaterally [11]. It is also important to acknowledge that cardiac arrhythmias in people with LQT2 can be misdiagnosed as seizures or epilepsy, which can lead to the initiation of unnecessary anti-seizure medications [57], which can further increase the risk of arrhythmic events and SCD [58]. In addition to better diagnosis, models that can help to comprehenssively evaluate the cardiac safety of anti-seizure medications will improve the medical management of people with LQTS.

This novel knock-in rabbit model of *Kcnh2*-mediated LQT2, epilepsy, and sudden death facilitates comprehensive studies to uncover the mechanisms underlying SCD and SUDEP in a population with both neuronal and cardiac electrical abnormalities.

### Limitations and future directions

Our RNAseq experiments provide a starting point to identify transcriptional diversity among WT and *Kcnh2*^(+/7bp−del)^ rabbits in the left ventricle and the brainstem. However, we note that analysis of these data is dependent upon the OryCun 2.0 genome assembly, which was last updated in 2014, and is poorly contiguous and poorly annotated when compared to more widely used model systems. This results in comparatively low rates of read mapping to coding regions. Future efforts to resolve this limitation will be valuable to transcriptome-wide analyses following *Kcnh2* 7bp deletion. Protein characterization was conducted using an antibody specific to full-length K_v_11.1^WT^. While this antibody demonstrates reduced K_v_11.1^WT^, which is in line with reduced WT *Kcnh2* expression, the antibody cannot detect mutant K_v_11.1 due to the frameshift and truncation. As many K_v_11.1 antibodies are raised in rabbits, particularly those upsteam of the 7bp-deletion, cross-reactivity prohibited assessments of total/mutant K_v_11.1 expression. Future studies will use myocytes and neurons from specific regions and layers of the brain and neuronal cell-types to examine the implications of the heterozygous *Kcnh2* mutation on Kv11.1 channel function and biophysical properties, as well as AP firing patterns/morphology in the heart and brain. All instances of neuronal and cardiac abnormalities reported in this manuscript were captured using our acute video-EEG-ECG recording system that uses subdermal pin electrodes [13]. While this setup facilities comprehenssive and high-quality recordings, due to the intermittent nature of these short-term recordings, we likely missed some instances of spontaneous epileptiform discharges, clinical seizures, arrhythmias, and the cascade of neuro-cardiac dysfunction leading up to spontaneous sudden death. Thus, we recently developed a long-term recording system that uses subdermally implanted electrodes to collect continuous 24/7 video-EEG-ECG recordings [59]. Future studies will provide more detailed insights into the prevalence, incidence, and physiological states of neuro-cardiac abnormalities in the rabbits. This novel knock-in rabbit model of LQT2 will enable us to perform extensive molecular, cellular, and in vivo studies to better understand the implications of *Kcnh2* variants throughout the body. This model is a valuable tool to investigate the cardiac safety of medications in an at-risk disease population.

## Conclusions

We developed a novel translational genetic rabbit model of *Kcnh2*-mediated LQT2, which reproduces the neuro-cardiac electrical abnormalities and sudden death seen in people with LQT2. *Kcnh2*^(+/7bp−del)^ rabbits exhibit altered *Kcnh2*/Kv11.1 expression, cardiac QT_c_ prolongation, ECG abnormalities, epileptic seizures, and sudden death. Knock-in *Kcnh2*^(+/7bp−del)^ rabbits that demonstrate multi-system pathologies serve as a great tool to study the mechanisms for and cascade leading up to sudden death (e.g., SCD & SUDEP). *Kcnh2*^(+/bp−del)^ rabbits provide a valuable translational model for the development of novel therapeutics and cardiac safety drug testing.

## Electronic supplementary material

Below is the link to the electronic supplementary material.

Supplementary Material 1

Supplementary Material 2

Supplementary Material 3

## Data Availability

The data that support the findings of this study are available to qualified investigators through data transfer and user agreements upon request from the corresponding author.

## Abbreviations

LQTS: Long QT Syndrome
SCD: Sudden Cardiac Death
ECG: Electrocardiogram
LQT2: Long QT Syndrome Type-2
LOF: Loss-of-function
AP: Action Potential
EEG: Electroencephalogram
LQT1: Long QT Syndrome Type-1
SUDEP: Sudden Unexpected Death in Epilepsy
ONT: Oxford Nanopore Technology
GTCS: Generalized tonic-clonic seizures
AV: Atrio-ventricular
PGES: Postictal Generalized EEG Suppression
PVC: Premature Ventricular Complex

## References

[1] Moss AJ, Kass RS. Long QT syndrome: from channels to cardiac arrhythmias. J Clin Invest. 2005;115(8):2018–24. 16075042 10.1172/JCI25537 PMC1180552

[2] Schwartz PJ, Stramba-Badiale M, Crotti L, Pedrazzini M, Besana A, Bosi G, et al. Prevalence of the congenital long-QT syndrome. Circulation. 2009;120(18):1761–7. 19841298 10.1161/CIRCULATIONAHA.109.863209 PMC2784143

[3] Moss AJ. Prolonged QT-interval syndromes. JAMA. 1986;256(21):2985–7. 3773216

[4] Zicha S, Moss I, Allen B, Varro A, Papp J, Dumaine R, et al. Molecular basis of species-specific expression of repolarizing K + currents in the heart. Am J Physiol Heart Circ Physiol. 2003;285(4):H1641–9. 12816752 10.1152/ajpheart.00346.2003

[5] Babcock JJ, Li M. hERG channel function: beyond long QT. Acta Pharmacol Sin. 2013;34(3):329–35. 23459091 10.1038/aps.2013.6 PMC3587915

[6] Trudeau MC, Warmke JW, Ganetzky B, Robertson GA. HERG, a human inward rectifier in the voltage-gated potassium channel family. Science. 1995;269(5220):92–5. 7604285 10.1126/science.7604285

[7] Thomas D, Karle CA, Kiehn J. The cardiac hERG/IKr potassium channel as pharmacological target: structure, function, regulation, and clinical applications. Curr Pharm Des. 2006;12(18):2271–83. 16787254 10.2174/138161206777585102

[8] Ji H, Tucker KR, Putzier I, Huertas MA, Horn JP, Canavier CC, et al. Functional characterization of ether-a-go-go-related gene potassium channels in midbrain dopamine neurons - implications for a role in depolarization block. Eur J Neurosci. 2012;36(7):2906–16. 22780096 10.1111/j.1460-9568.2012.08190.x PMC4042402

[9] Auerbach DS, McNitt S, Gross RA, Zareba W, Dirksen RT, Moss AJ. Genetic biomarkers for the risk of seizures in long QT syndrome. Neurology. 2016;87(16):1660–8. 27466471 10.1212/WNL.0000000000003056 PMC5085072

[10] Anderson JH, Bos JM, Cascino GD, Ackerman MJ. Prevalence and spectrum of electroencephalogram-identified epileptiform activity among patients with long QT syndrome. Heart Rhythm. 2014;11(1):53–7. 24103226 10.1016/j.hrthm.2013.10.010

[11] Gonzalez A, Aurlien D, Larsson PG, Olsen KB, Dahl IT, Edvardsen T, et al. Seizure-like episodes and EEG abnormalities in patients with long QT syndrome. Seizure. 2018;61:214–20. 30218808 10.1016/j.seizure.2018.08.020

[12] Bagnall RD, Crompton DE, Semsarian C. Genetic Basis of Sudden Unexpected Death in Epilepsy. Front Neurol. 2017;8:348. 28775708 10.3389/fneur.2017.00348 PMC5517398

[13] Bosinski C, Wagner K, Zhou X, Liu L, Auerbach DS. Multi-system Monitoring for Identification of Seizures, Arrhythmias and Apnea in Conscious Restrained Rabbits. J Vis Exp. 2021(169). 10.3791/62256 33843929

[14] Nattel S, Duker G, Carlsson L. Model systems for the discovery and development of antiarrhythmic drugs. Prog Biophys Mol Biol. 2008;98(2–3):328–39. 19038282 10.1016/j.pbiomolbio.2008.10.009

[15] Moss AJ, Zareba W, Benhorin J, Locati EH, Hall WJ, Robinson JL, et al. ECG T-wave patterns in genetically distinct forms of the hereditary long QT syndrome. Circulation. 1995;92(10):2929–34. 7586261 10.1161/01.cir.92.10.2929

[16] Zhang L, Timothy KW, Vincent GM, Lehmann MH, Fox J, Giuli LC, et al. Spectrum of ST-T-wave patterns and repolarization parameters in congenital long-QT syndrome: ECG findings identify genotypes. Circulation. 2000;102(23):2849–55. 11104743 10.1161/01.cir.102.23.2849

[17] Porta-Sanchez A, Spillane DR, Harris L, Xue J, Dorsey P, Care M, et al. T-Wave Morphology Analysis in Congenital Long QT Syndrome Discriminates Patients From Healthy Individuals. JACC Clin Electrophysiol. 2017;3(4):374–81. 29759450 10.1016/j.jacep.2016.10.013

[18] Yamada T, Yoshida N, Itoh T, Litovsky SH, Doppalapudi H, McElderry HT et al. Idiopathic Ventricular Arrhythmias Originating From the Parietal Band: Electrocardiographic and Electrophysiological Characteristics and Outcome of Catheter Ablation. Circ Arrhythm Electrophysiol. 2017;10(8). 10.1161/CIRCEP.117.005099 28794085

[19] Soylemez N, Yaman B. Association between ventricular premature contraction burden and ventricular repolarization duration. Rev Assoc Med Bras (1992). 2022;68(11):1571–5. 36449776 10.1590/1806-9282.20220676 PMC9720762

[20] Kural MA, Duez L, Sejer Hansen V, Larsson PG, Rampp S, Schulz R, et al. Criteria for defining interictal epileptiform discharges in EEG: A clinical validation study. Neurology. 2020;94(20):e2139–47. 32321764 10.1212/WNL.0000000000009439 PMC7526669

[21] Schwartz PJ, Priori SG, Spazzolini C, Moss AJ, Vincent GM, Napolitano C, et al. Genotype-phenotype correlation in the long-QT syndrome: gene-specific triggers for life-threatening arrhythmias. Circulation. 2001;103(1):89–95. 11136691 10.1161/01.cir.103.1.89

[22] Crotti L. From gene-specific to function-specific risk stratification in long QT syndrome Type 2: implications for clinical management. EP Europace. 2023;25(4):1320–2. 10.1093/europace/euad035 PMC10105882 36857538

[23] Aizawa T, Wada Y, Hasegawa K, Huang H, Imamura T, Gao J, et al. Non-missense variants of KCNH2 show better outcomes in type 2 long QT syndrome. Europace. 2023;25(4):1491–9. 36861347 10.1093/europace/euac269 PMC10105889

[24] Tu E, Bagnall RD, Duflou J, Semsarian C. Post-mortem review and genetic analysis of sudden unexpected death in epilepsy (SUDEP) cases. Brain Pathol. 2011;21(2):201–8. 20875080 10.1111/j.1750-3639.2010.00438.x PMC8094243

[25] Casini S, Verkerk AO, Remme CA. Human iPSC-Derived Cardiomyocytes for Investigation of Disease Mechanisms and Therapeutic Strategies in Inherited Arrhythmia Syndromes: Strengths and Limitations. Cardiovasc Drugs Ther. 2017;31(3):325–44. 28721524 10.1007/s10557-017-6735-0 PMC5550530

[26] London B. Cardiac arrhythmias: from (transgenic) mice to men. J Cardiovasc Electrophysiol. 2001;12(9):1089–91. 11573703 10.1046/j.1540-8167.2001.01089.x

[27] Nerbonne JM. Studying cardiac arrhythmias in the mouse–a reasonable model for probing mechanisms? Trends Cardiovasc Med. 2004;14(3):83–93. 15121155 10.1016/j.tcm.2003.12.006

[28] Nerbonne JM, Nichols CG, Schwarz TL, Escande D. Genetic manipulation of cardiac K(+) channel function in mice: what have we learned, and where do we go from here? Circ Res. 2001;89(11):944–56. 11717150 10.1161/hh2301.100349

[29] Edwards AG, Louch WE. Species-Dependent Mechanisms of Cardiac Arrhythmia: A Cellular Focus. Clin Med Insights Cardiol. 2017;11:1179546816686061. 28469490 10.1177/1179546816686061 PMC5392019

[30] Babij P, Askew GR, Nieuwenhuijsen B, Su CM, Bridal TR, Jow B, et al. Inhibition of cardiac delayed rectifier K + current by overexpression of the long-QT syndrome HERG G628S mutation in transgenic mice. Circ Res. 1998;83(6):668–78. 9742063 10.1161/01.res.83.6.668

[31] Lees-Miller JP, Guo J, Somers JR, Roach DE, Sheldon RS, Rancourt DE, et al. Selective knockout of mouse ERG1 B potassium channel eliminates I(Kr) in adult ventricular myocytes and elicits episodes of abrupt sinus bradycardia. Mol Cell Biol. 2003;23(6):1856–62. 12612061 10.1128/MCB.23.6.1856-1862.2003 PMC149456

[32] Teng GQ, Zhao X, Lees-Miller JP, Quinn FR, Li P, Rancourt DE, et al. Homozygous missense N629D hERG (KCNH2) potassium channel mutation causes developmental defects in the right ventricle and its outflow tract and embryonic lethality. Circ Res. 2008;103(12):1483–91. 18948620 10.1161/CIRCRESAHA.108.177055 PMC2774899

[33] Teng G, Zhao X, Lees-Miller JP, Belke D, Shi C, Chen Y, et al. Role of mutation and pharmacologic block of human KCNH2 in vasculogenesis and fetal mortality: partial rescue by transforming growth factor-beta. Circ Arrhythm Electrophysiol. 2015;8(2):420–8. 25648353 10.1161/CIRCEP.114.001837

[34] Browning RA, Nelson DK. Variation in threshold and pattern of electroshock-induced seizures in rats depending on site of stimulation. Life Sci. 1985;37(23):2205–11. 4068901 10.1016/0024-3205(85)90573-9

[35] Skradski SL, Clark AM, Jiang H, White HS, Fu YH, Ptacek LJ. A novel gene causing a mendelian audiogenic mouse epilepsy. Neuron. 2001;31(4):537–44. 11545713 10.1016/s0896-6273(01)00397-x

[36] Trosclair K, Dhaibar HA, Gautier NM, Mishra V, Glasscock E. Neuron-specific Kv1.1 deficiency is sufficient to cause epilepsy, premature death, and cardiorespiratory dysregulation. Neurobiol Dis. 2020;137:104759. 31978607 10.1016/j.nbd.2020.104759 PMC7050436

[37] Xiong Y, Mahmood A, Chopp M. Animal models of traumatic brain injury. Nat Rev Neurosci. 2013;14(2):128–42. 23329160 10.1038/nrn3407 PMC3951995

[38] Ellermann C, Wolfes J, Eckardt L, Frommeyer G. Role of the rabbit whole-heart model for electrophysiologic safety pharmacology of non-cardiovascular drugs. Europace. 2021;23(6):828–36. 33200170 10.1093/europace/euaa288

[39] Aubert M, Osterwalder R, Wagner B, Parrilla I, Cavero I, Doessegger L, et al. Evaluation of the rabbit Purkinje fibre assay as an in vitro tool for assessing the risk of drug-induced torsades de pointes in humans. Drug Saf. 2006;29(3):237–54. 16524323 10.2165/00002018-200629030-00007

[40] Roche M, Renauleaud C, Ballet V, Doubovetzky M, Guillon JM. The isolated rabbit heart and Purkinje fibers as models for identifying proarrhythmic liability. J Pharmacol Toxicol Methods. 2010;61(3):238–50. 20117224 10.1016/j.vascn.2010.01.011

[41] Brunner M, Peng X, Liu GX, Ren XQ, Ziv O, Choi BR, et al. Mechanisms of cardiac arrhythmias and sudden death in transgenic rabbits with long QT syndrome. J Clin Invest. 2008;118(6):2246–59. 18464931 10.1172/JCI33578 PMC2373420

[42] Anson BD, Ackerman MJ, Tester DJ, Will ML, Delisle BP, Anderson CL, et al. Molecular and functional characterization of common polymorphisms in HERG (KCNH2) potassium channels. Am J Physiol Heart Circ Physiol. 2004;286(6):H2434–41. 14975928 10.1152/ajpheart.00891.2003

[43] Khan IA, Long. QT syndrome: diagnosis and management. Am Heart J. 2002;143(1):7–14. 11773906 10.1067/mhj.2002.120295

[44] Hirdes W, Napp N, Wulfsen I, Schweizer M, Schwarz JR, Bauer CK. Erg K + currents modulate excitability in mouse mitral/tufted neurons. Pflugers Arch. 2009;459(1):55–70. 19688350 10.1007/s00424-009-0709-4

[45] Chiesa N, Rosati B, Arcangeli A, Olivotto M, Wanke E. A novel role for HERG K + channels: spike-frequency adaptation. J Physiol. 1997;501(Pt 2):313–8. 9192303 10.1111/j.1469-7793.1997.313bn.x PMC1159479

[46] Najm I, Ying Z, Janigro D. Mechanisms of epileptogenesis. Neurol Clin. 2001;19(2):237–50. 11358743 10.1016/s0733-8619(05)70017-7

[47] Bendall JR. The shortening of rabbit muscles during rigor mortis; its relation to the breakdown of adenosine triphosphate and creatine phosphate and to muscular contraction. J Physiol. 1951;114(1–2):71–88. 14861784 10.1113/jphysiol.1951.sp004604 PMC1392100

[48] Singh V, Ryan JM, Auerbach DS. It is premature for a unified hypothesis of sudden unexpected death in epilepsy: A great amount of research is still needed to understand the multisystem cascade. Epilepsia. 2023;64(8):2006–10. 37129136 10.1111/epi.17636

[49] Ryvlin P, Nashef L, Lhatoo SD, Bateman LM, Bird J, Bleasel A, et al. Incidence and mechanisms of cardiorespiratory arrests in epilepsy monitoring units (MORTEMUS): a retrospective study. Lancet Neurol. 2013;12(10):966–77. 24012372 10.1016/S1474-4422(13)70214-X

[50] Vedovello M, Baldacci F, Nuti A, Cipriani G, Ulivi M, Vergallo A, et al. Peri-ictal prolonged atrial fibrillation after generalized seizures: description of a case and etiopathological considerations. Epilepsy Behav. 2012;23(3):377–8. 22341957 10.1016/j.yebeh.2012.01.005

[51] Vilella L, Miyake CY, Chaitanya G, Hampson JP, Omidi SJ, Ochoa-Urrea M, et al. Incidence and Types of Cardiac Arrhythmias in the Peri-Ictal Period in Patients Having a Generalized Convulsive Seizure. Neurology. 2024;103(1):e209501. 38870452 10.1212/WNL.0000000000209501 PMC11759939

[52] Logroscino G, Hesdorffer DC, Cascino G, Annegers JF, Hauser WA. Short-term mortality after a first episode of status epilepticus. Epilepsia. 1997;38(12):1344–9. 9578531 10.1111/j.1528-1157.1997.tb00073.x

[53] Partemi S, Vidal MC, Striano P, Campuzano O, Allegue C, Pezzella M, et al. Genetic and forensic implications in epilepsy and cardiac arrhythmias: a case series. Int J Legal Med. 2015;129(3):495–504. 25119684 10.1007/s00414-014-1063-4

[54] Soh MS, Bagnall RD, Bennett MF, Bleakley LE, Mohamed Syazwan ES, Phillips AM, et al. Loss-of-function variants in K(v) 11.1 cardiac channels as a biomarker for SUDEP. Ann Clin Transl Neurol. 2021;8(7):1422–32. 34002542 10.1002/acn3.51381 PMC8283159

[55] Chahal CAA, Gottwald JA, St Louis EK, Xie J, Brady PA, Alhurani RE, et al. QT prolongation in patients with index evaluation for seizure or epilepsy is predictive of all-cause mortality. Heart Rhythm. 2022;19(4):578–84. 34775068 10.1016/j.hrthm.2021.11.013 PMC8977248

[56] Partemi S, Cestele S, Pezzella M, Campuzano O, Paravidino R, Pascali VL, et al. Loss-of-function KCNH2 mutation in a family with long QT syndrome, epilepsy, and sudden death. Epilepsia. 2013;54(8):e112–6. 23899126 10.1111/epi.12259

[57] Kang H, Lan L, Jia Y, Li C, Fang Y, Zhu S, et al. Long QT syndrome with potassium voltage-gated channel subfamily H member 2 gene mutation mimicking refractory epilepsy: case report. BMC Neurol. 2021;21(1):338. 34481479 10.1186/s12883-021-02365-8 PMC8418736

[58] Auerbach DS, Biton Y, Polonsky B, McNitt S, Gross RA, Dirksen RT, et al. Risk of cardiac events in Long QT syndrome patients when taking antiseizure medications. Transl Res. 2018;191:81–92. e7. 29121487 10.1016/j.trsl.2017.10.002 PMC5733703

[59] Williams LG, Wagner KT, Samaniego N, Singh V, Ryan JM, Auerbach DS. Surgical Implant Procedure and Wiring Configuration for Continuous Long-Term EEG/ECG Monitoring in Rabbits. J Vis Exp. 2025(215). 10.3791/67620 PMC11951853 39927654

