# Supplementary material for: Knock-in Kcnh2 rabbit model of long QT syndrome type-2, epilepsy, and sudden death
Source: J Transl Med. 2025 Apr 15;23:446. doi: 10.1186/s12967-025-06382-w (PMC12001650; doi:10.1186/s12967-025-06382-w)
Supplement: Supplementary file 3 — Supplementary Material 3 [file 12967_2025_6382_MOESM3_ESM.pdf]

WT Rabbit Normal EEG-ECG (Awake)

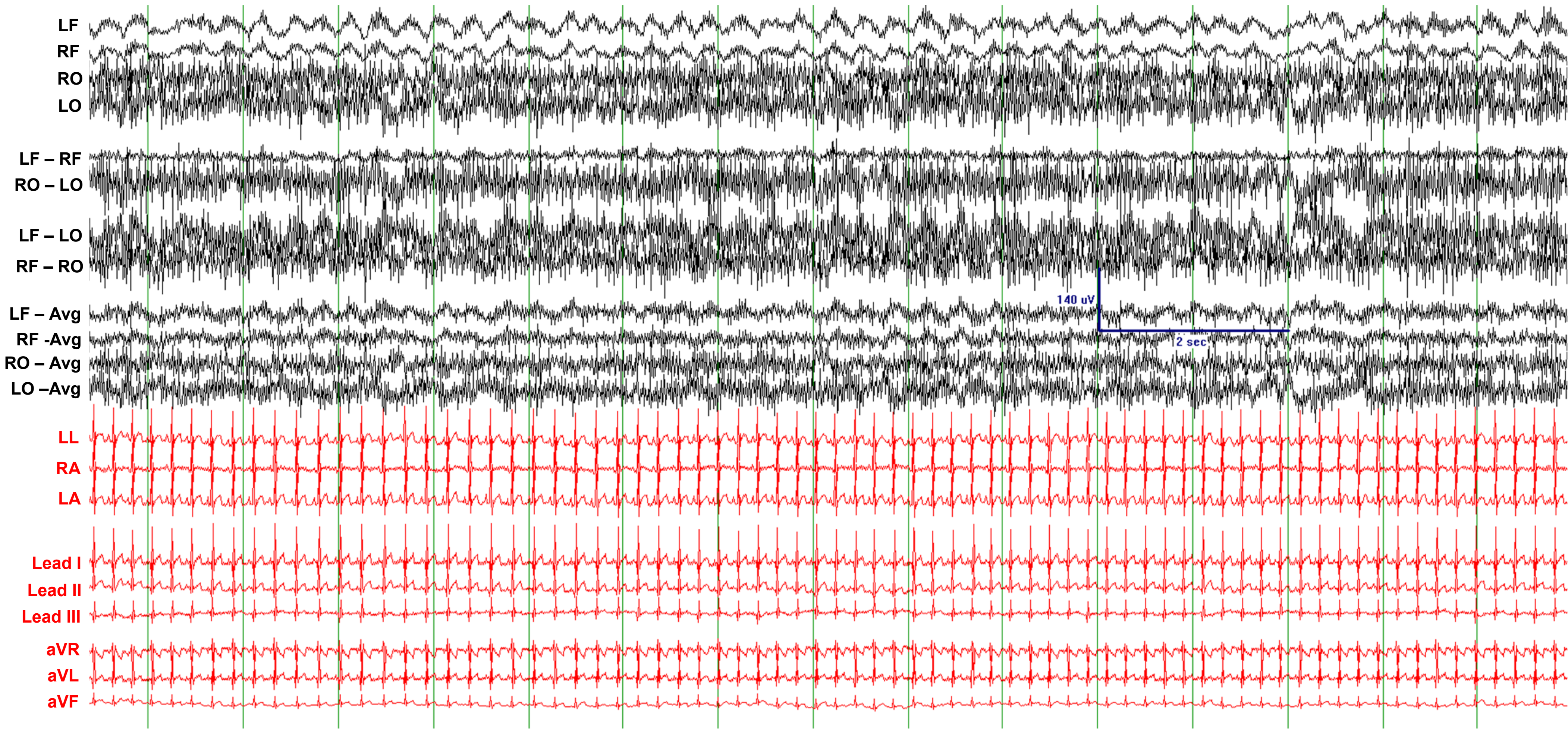

Supplementary Fig. 1: Baseline EEG (black) and ECG (red) in a 1.5-month-old male WT rabbit. EEG: Referential, bipolar, and unipolar traces. ECG: Referential, bipolar limb (I, II, III), and augmented (aVR, aVL, aVF) lead configurations. Scale bar indicates EEG=140μV and 2 seconds.

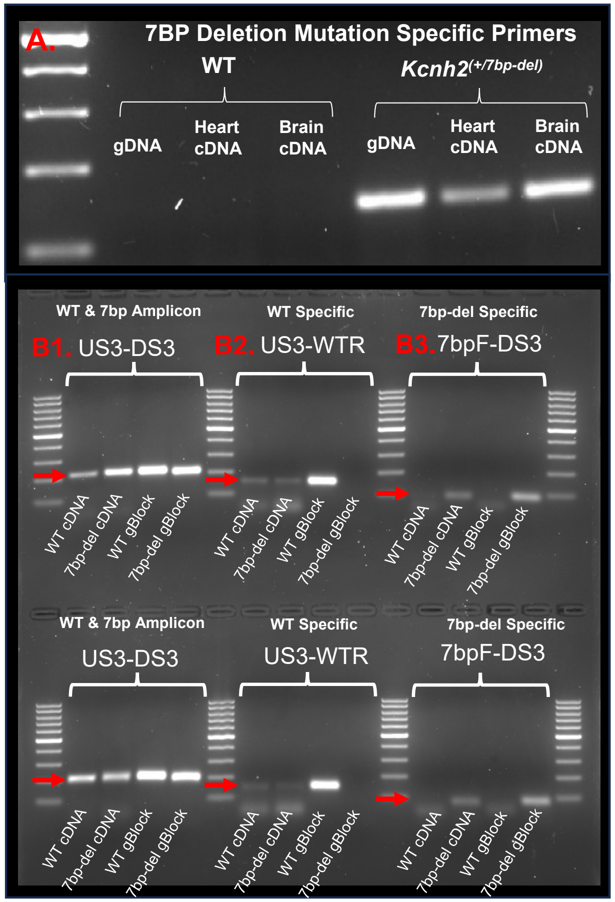

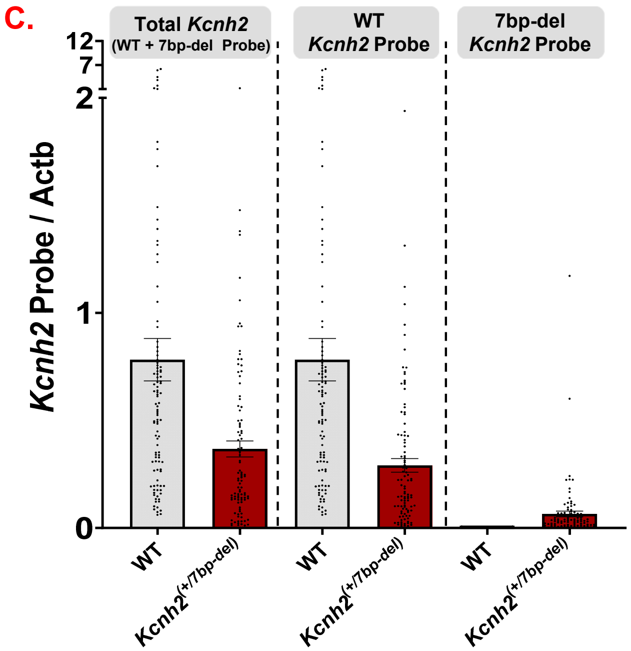

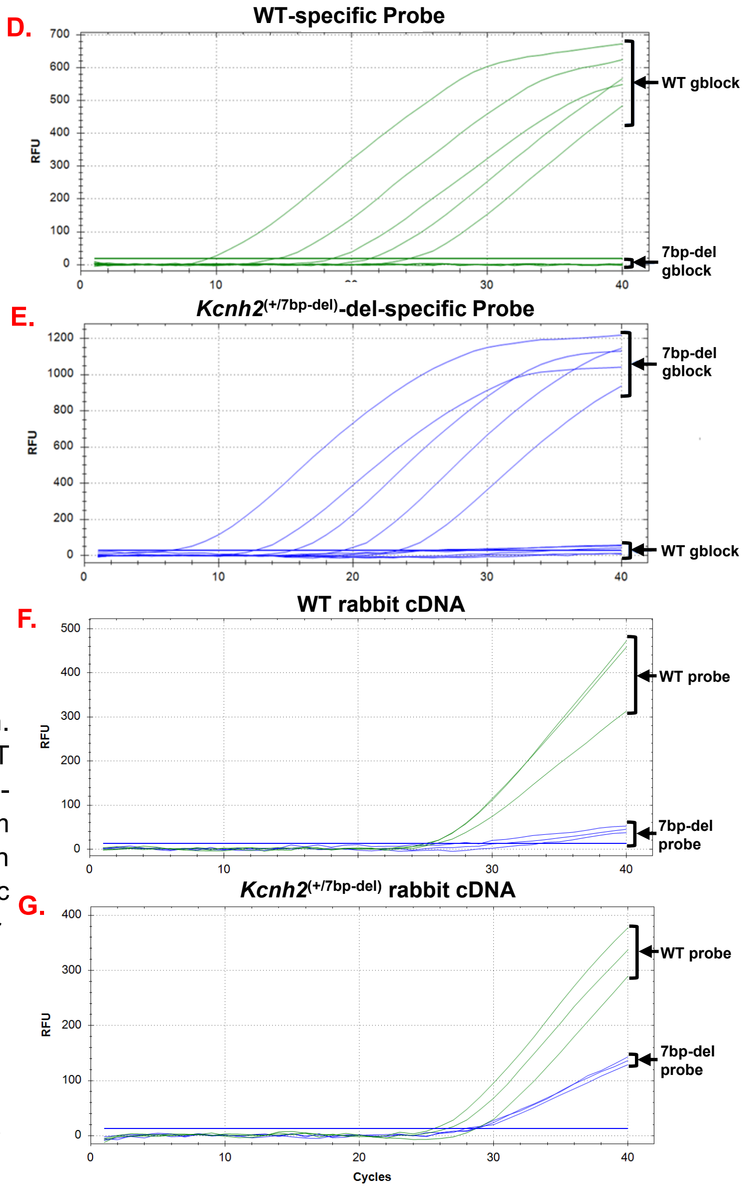

Supplementary Fig. 2: qPCR primer and probe verification. **A.** PCR of genomic DNA and complimentary DNA of the WT and *Kcnh2*$^{(+/7bp\text{-}del)}$ heart and brain tissue, using a mutation-specific primer. **B1.** *Kcnh2* upstream (US3) and downstream (DS3) primers amplify 216bp region in WT and 209bp region in 7bp-del cDNA and synthetic DNA (g-block). **B2.** WT-specific primer amplifies 162bp region in WT and 7bp-del cDNA and WT synthetic DNA. **B3.** 7bp-del-specific primer amplifies 89bp region in 7bp-del cDNA and synthetic DNA. WTR: WT-reverse; 7bpF: 7bp-del-forward. **C.** Relative gene expression of total *Kcnh2* (WT + mutant), WT *Kcnh2*, and 7bp-del mutant *Kcnh2* transcripts grouped across fifteen regions in WT and mutant tissue (WT: N=7 rabbits; 7bp: N=7 rabbits). **D.** Amplification plots of probe-based qPCR. WT-specific probe with WT synthetic DNA and 7bp-del mutant synthetic DNA. **E.** 7bp-del-specific probe with WT synthetic DNA and 7bp-del mutant synthetic DNA. **F.** cDNA from WT rabbit tissue with WT-specific probe (green) and 7bp-del-specific probe (blue). **G.** cDNA from *Kcnh2*$^{(+/7bp\text{-}del)}$ rabbit tissue with WT-specific probe (green) and 7bp-del-specific probe (blue).

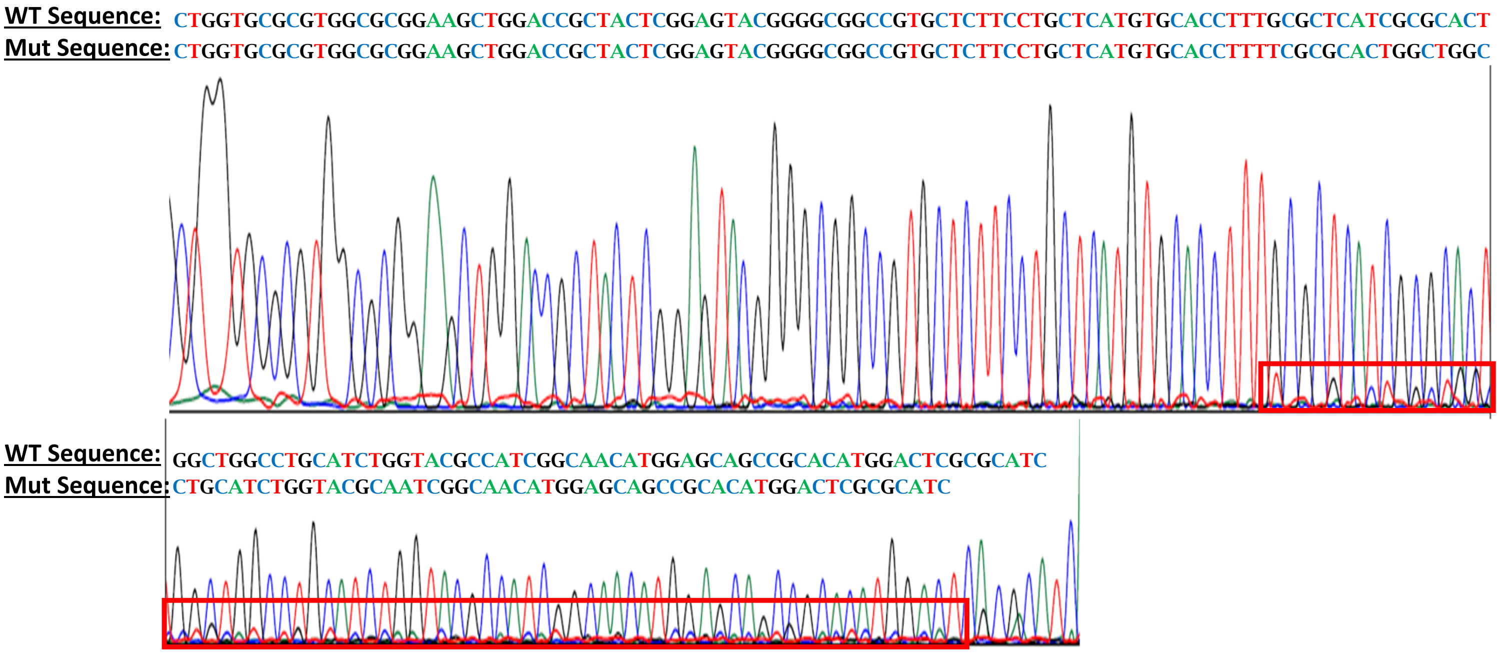

Supplementary Fig. 3: Sanger Sequencing Electropherogram. qPCR primers used in *Kcnh2*$^{(+/7bp\text{-}del)}$ cDNA. Primers used: US3, DS3.

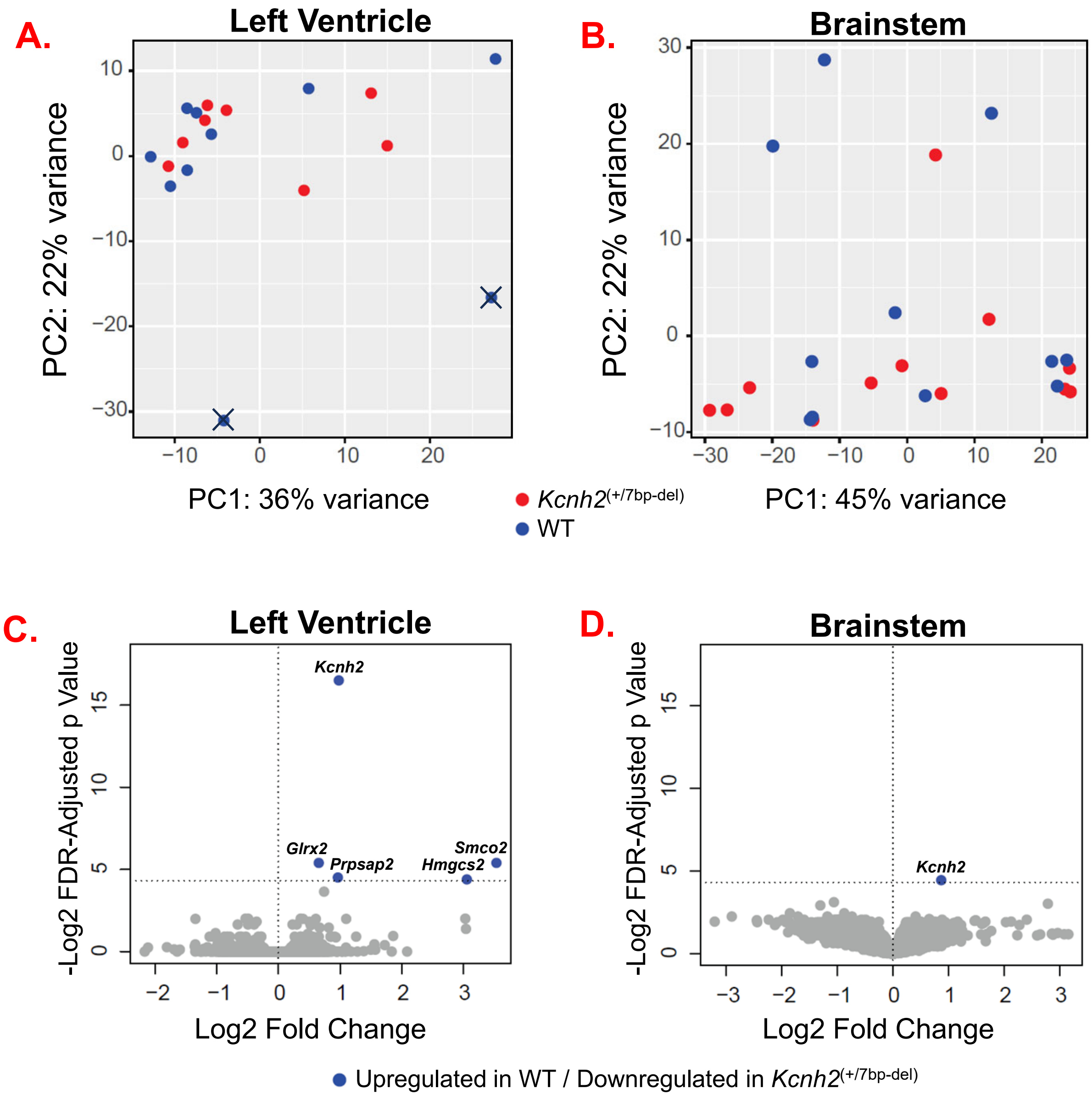

Supplementary Fig. 4: Bulk tissue RNA sequencing. PCA identifies transcriptional variability across samples, color-coded by genotype, for **A.** left ventricle and **B.** brainstem datasets. *Note: Crossed-out dots denote left ventricle samples that were relatively isolated in PC space with extreme values along PC2; these samples were removed from further analysis.* Differentially expressed genes across genotypes were identified using DESeq2; sex and total counts were included as covariates in the model. Results are depicted in separate volcano plots for **C.** left ventricle and **D.** brainstem datasets. Genes showing an adjusted p value (Benjamini-Hochberg) <0.05 were identified as significant. Left ventricle samples (WT N=8; *Kcnh2*$^{(+/7bp\text{-}del)}$ N=8); Brainstem samples (WT N=11; *Kcnh2*$^{(+/7bp\text{-}del)}$ N=12).

Primer Description (NM_001082384.1)	Primer Sequence	Location
Genotyping Primers		
**Rabbit *Kcnh2*-specific forward primer (GenF1)**	5’ ctggcgtgggacgtgcttca 3’	Intron 5
**Total and mutant rabbit *Kcnh2* common reverse primer (GenR1)**	5’ aggccgctgctgttgtagg 3’	Exon 7
**7bp-specific rabbit *Kcnh2* forward primer**	5’ ttgctcatgtgcaccttttcgcg 3’	Exon 7
qPCR Primers		
**Forward primer for rabbit *Kcnh2* (US3)**	5’ atcttcggctctggctctgag 3’	Exon 6
**Reverse primer for rabbit *Kcnh2* (DS3)**	5’ gatgcgcgagtccatgtg 3’	Exon 7
**Forward primer for rabbit *Actb***	5’ atgtgcaaggccggctt 3’	Exon 2
**Reverse primer for rabbit Actb**	5’ acgatgccgtgctcgat 3’	Exon 3
ONT Sequencing Primers		
**Forward primer for rabbit *Kcnh2***	5’ ctgccttcttgctgaag 3’	Exon 6
**Reverse primer for rabbit *Kcnh2***	5’ agaagatcttctccgagttg 3’	Exon 7
**Probe Description**	**Probe Sequence**	
ONT Sequencing Probes		
**WT-specific Kcnh2 probe sequence**	/56-FAM/agccagtgc/ZEN/gcgatgagc	
**Mutant-specific (7bp) Kcnh2 probe sequence**	/5HEX/ ttgctcatg/ZEN/tgcaccttttcgcg	
K_v11.1 Antibody		
**K_v11.1 C-terminus epitope amino acid sequence**	CGALTSQPLHRHGSDPGS	

Supplementary Table 1: Sequences of all primers and probes used for PCR, qPCR, and ONT sequencing. Amino acid sequence of anti-K_v11.1.

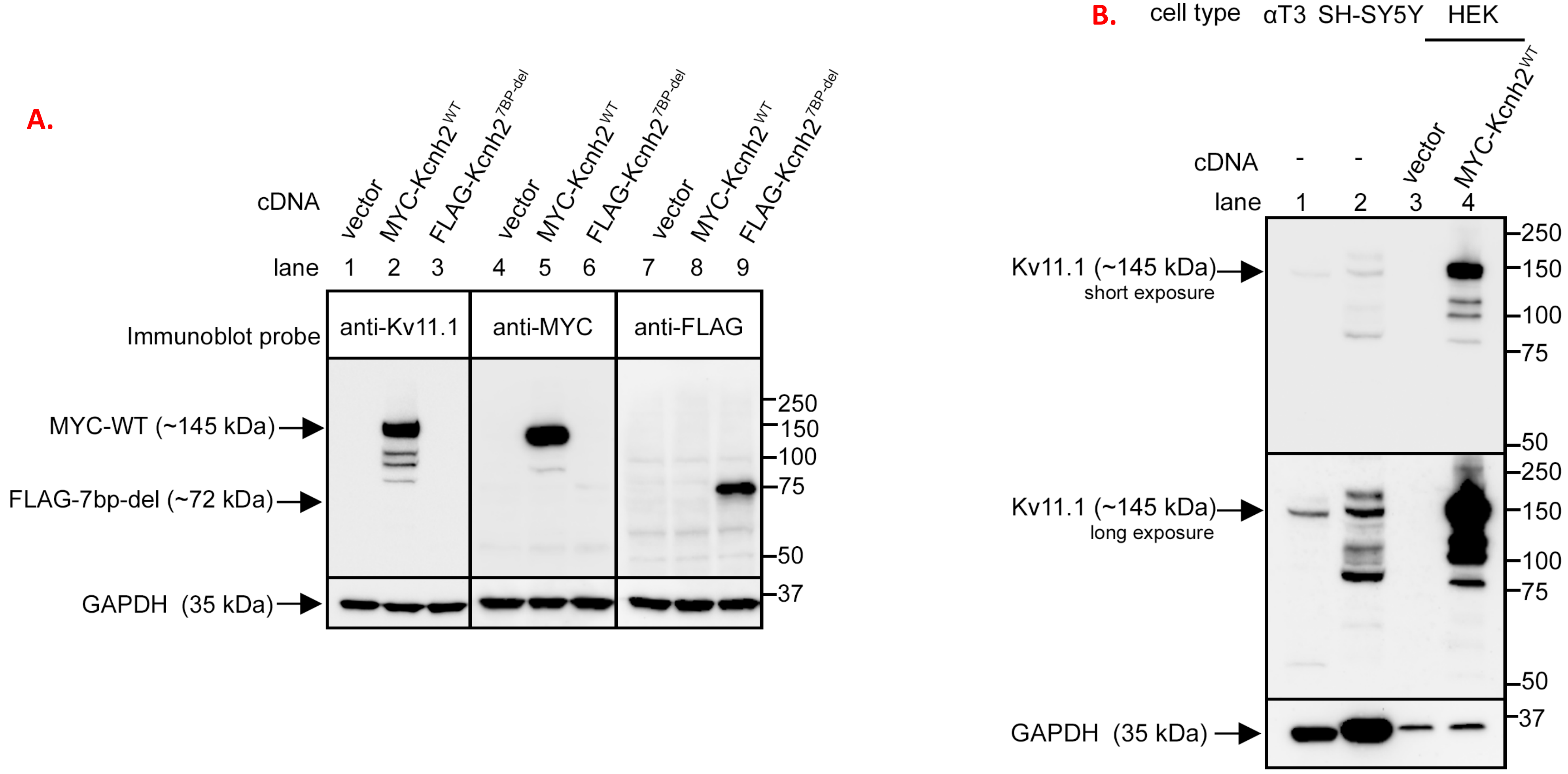

Supplementary Fig. 5: A. Immunoblot showing immunoreactivity of anti-K_v11.1 and anti-MYC in recognizing myc-$K_v11.1^{WT}$ and anti-FLAG in recognizing $K_v11.1^{7bp\text{-}del}$ constructs over-expressed in HEK cells. **B.** Immunoblot showing immunoreactivity of anti-K_v11.1 in recognizing endogenously expressed K_v11.1 in mouse pituitary (α-T3) and human neuroblastoma (SH-SY5Y) cell lines.

	Wild Type	7bp-del	Statistical Analyses		
			Wilcoxon Rank Sum	Logistic Regression	
**ECG Measure**	**Mean ± Standard Deviation**	**Mean ± Standard Deviation**	**p-value**	**Odds Ratio (95% CI)**	**p-value**
**Heart Rate**	273.91 ± 52.052	287.04 ± 53.10	0.164767396	0.988 (0.979,0.997)	**0.010008996**
**P Duration**	25.47 ± 4.37	26.08 ± 5.48	0.891218847	0.967 (0.863,1.082)	0.554669121
**PR**	59.21 ± 10.23	54.84 ± 8.56	**0.018110803**	1.099 (1.019,1.185)	**0.014812847**
**QT_c**	244.51 ± 18.82	279.14 ± 21.75	**2.77743×10^{-14}**	0.913 (0.882,0.944)	**1.5225×10^{-7}**
**QRS**	25.09 ± 5.21	24.84 ± 5.09	0.605954274	1.022 (0.899,1.161)	0.742333248
**JT_{ec}**	193.98 ± 16.64	227.41 ± 19.03	**1.95451×10^{-15}**	0.895 (0.86,0.932)	**5.79916×10^{-8}**
**JT_{pc}**	137.91 ± 21.91	180.46 ± 19.51	**3.22658×10^{-15}**	0.919 (0.893,0.947)	**1.88463×10^{-8}**
**T_pT_e**	27.74 ± 9.57	22.40 ± 5.86	**0.002081525**	1.104 (1.037,1.174)	**0.00188449**

Supplementary Table 2: Conduction and repolarization ECG metrics in WT and *Kcnh2*$^{(+/7bp\text{-}del)}$ rabbits. Wilcoxon rank sum test; logistic regression model adjusts for age, sex, and heart rate.

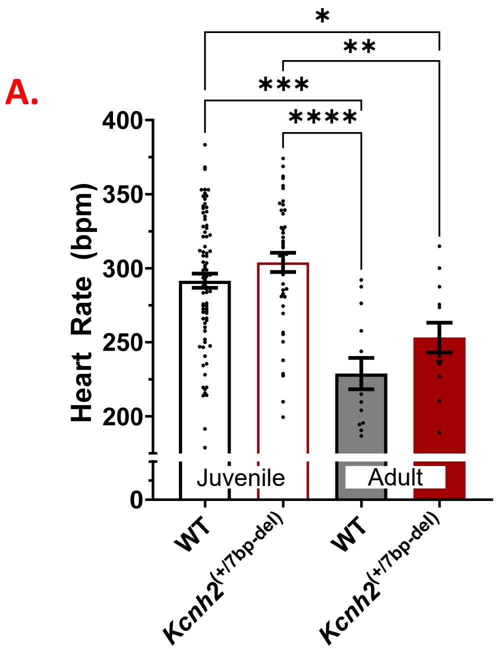

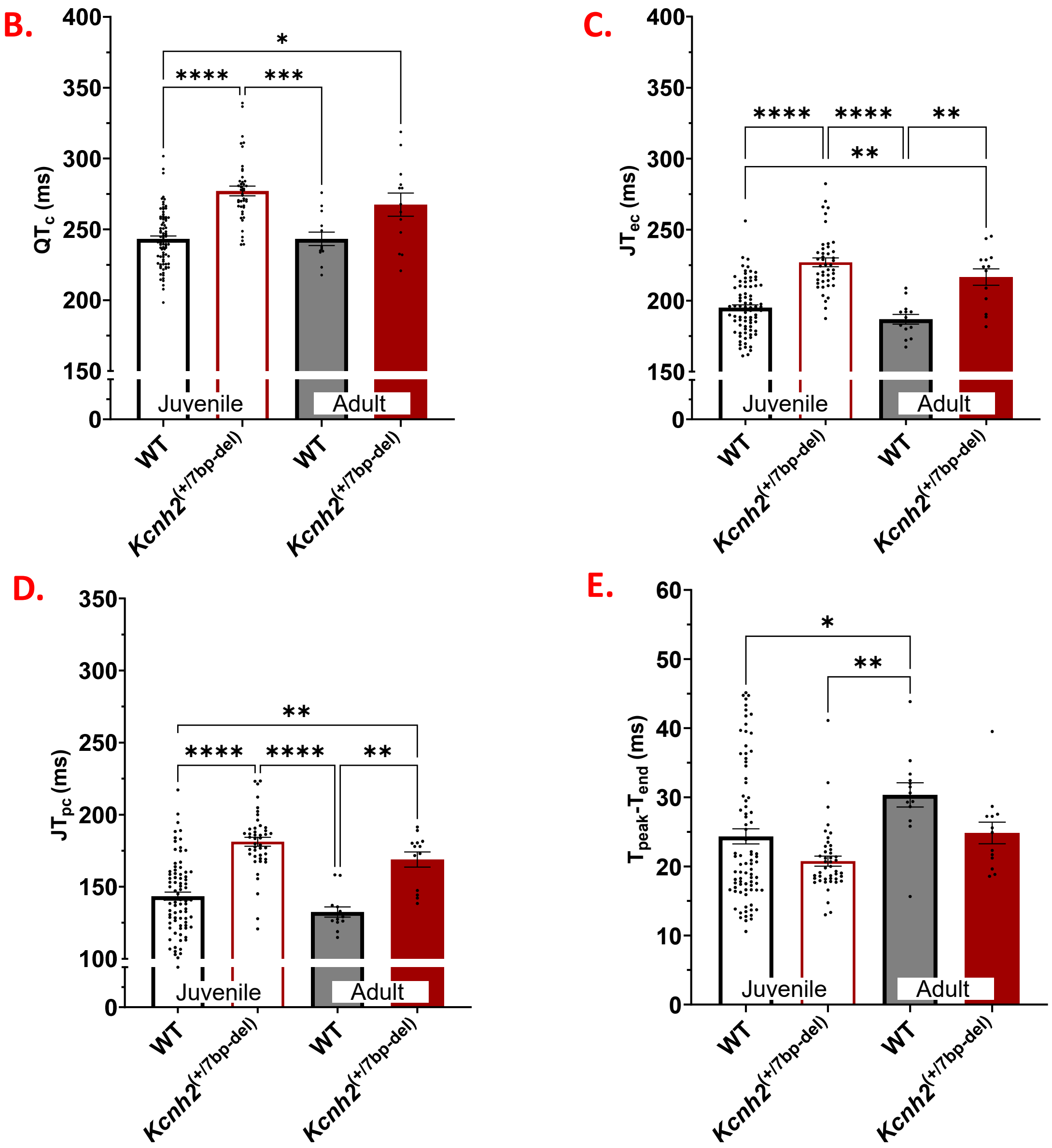

Supplementary Fig. 6: Genotype-specific ECG measures at juvenile (<3 months) and adult (>6months) timepoints. Each point is a 5-minute period of the recording. WT (N=68, n=89), *Kcnh2*$^{(+/7bp\text{-}del)}$ (N=37, n=52).

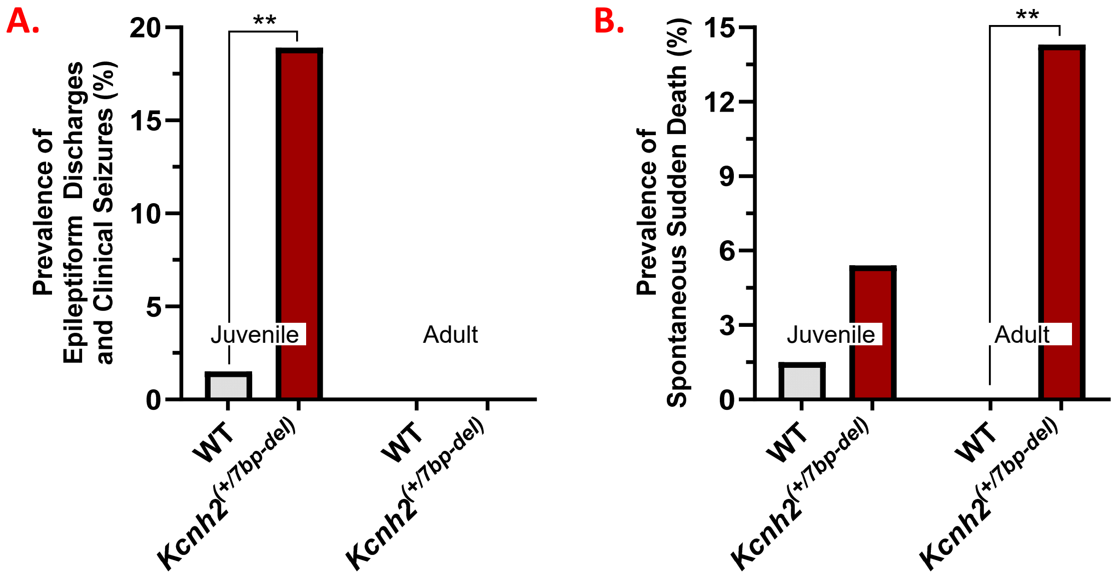

Supplementary Fig. 7: Prevalence of spontaneous **A.** epileptiform discharges and clinical seizures (WT N=68 rabbits, n=140 recordings; *Kcnh2*$^{(+/7bp\text{-}del)}$ N=37, n=85), and **B.** sudden death (WT N=68 rabbits; *Kcnh2*$^{(+/7bp\text{-}del)}$ N=37) at juvenile (<3 months) and adult (>6months) timepoints. Statistical analyses: performed Fisher's exact test. **, p<0.01.

A. QT$_c$: Baseline (All Littermates)

	N	Median (ms)	Standard Deviation (ms)	Wilcoxon p-value
**WT**	2	263.3	21.6	<0.0001
**7bp-del**	4	310.7	7.4	

B. QT$_c$: Percent change in QT$_c$ in the *Kcnh2*$^{(+/7bp\text{-}del)}$ Sudden Death Case-1 on the lethal vs. non-lethal days

	Median (%)	Standard Deviation (%)	Signed-Rank p-value
**7bp-del**	10.3	7.2	<0.0001

Supplementary Table 3: Cardiac QT$_c$ duration in Sudden Death Case-1 and its littermates: **A.** Baseline QT$_c$ recorded 2-days prior to sudden death in 3 *Kcnh2*$^{(7bp\text{-}del)}$ (one is Sudden Death Case 1) and 2 WT littermates. **B**. Percent change in QT$_c$ in the *Kcnh2*$^{(+/7bp\text{-}del)}$ Sudden Death Case-1 on the lethal vs. non-lethal days.

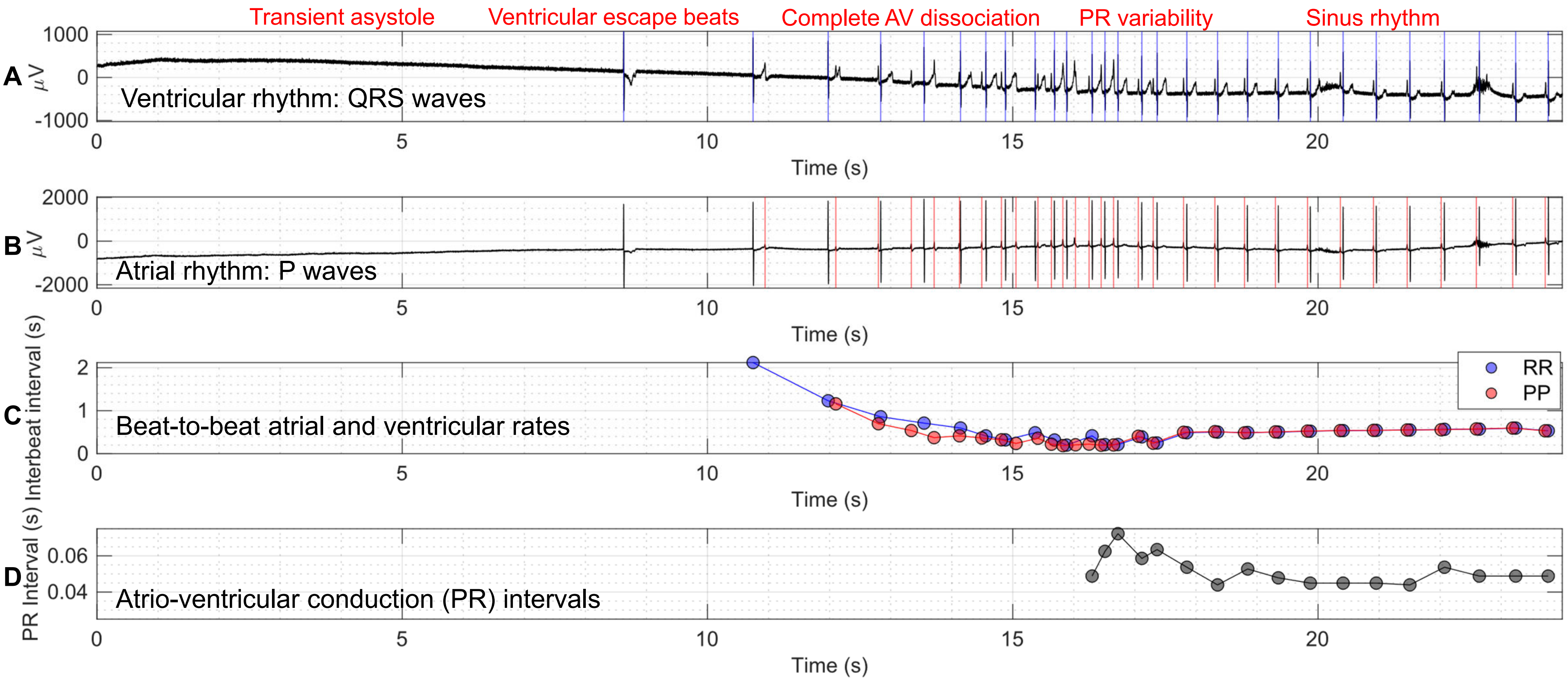

Supplementary Fig. 8: Progression of cardiac abnormalities and recovery following a convulsive seizure. **A.** Ventricular rhythm: ECG referential lead LL with each QRS complex annotated. **B.** Atrial rhythm: ECG referential lead LA with each P wave annotated. **C.** Instantaneous rates: Ventricular (RR) and atrial (PP) beat-to-beat intervals. **D.** Atrio-ventricular conduction: PR intervals for all sinus beats plotted over time. LL: left leg, LA: left arm.

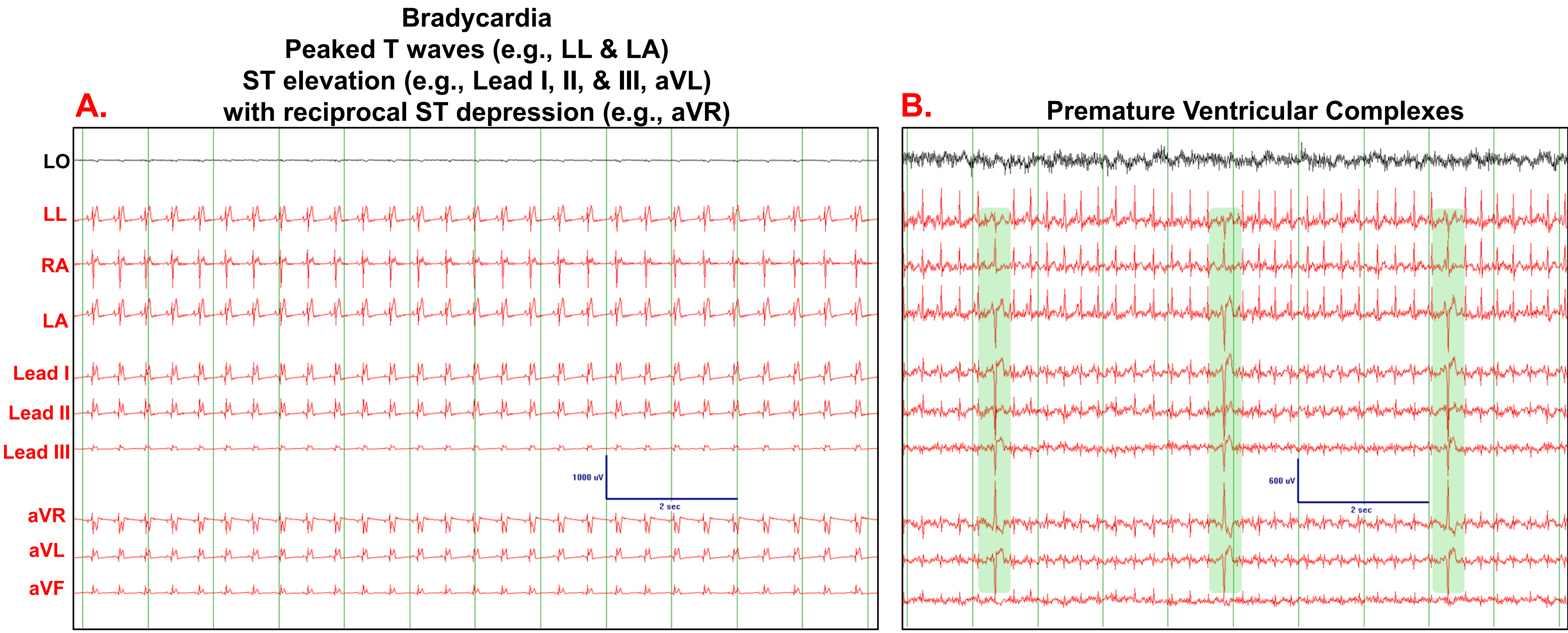

Supplementary Fig. 9: Cardiac abnormalities noted in $\textit{Kcnh2}^{(+/7bp\text{-}del)}$ rabbits, depicted using referential and standard (I, II, III, and augmented) ECG lead configurations. **A.** Peaked T waves in referential leads (LA, LL). ST elevation in leads I, II, and aVL and reciprocal ST depression in aVR. Sudden death Case 2: $\textit{Kcnh2}^{(+/7bp\text{-}del)}$ male 7-week-old rabbit **B.** PVC: Premature Ventricular Complex. Sudden Death Case 5: $\textit{Kcnh2}^{(+/7bp\text{-}del)}$ female 13-month-old rabbit. ECG scale bar indicates 1000μV (A) and 600μV amplitude (B) and 2 seconds. EEG scale: 140μV amplitude.

Kcnh2$^{(+/7bp\text{-}del)}$ 7-week-old Sudden Cardiac Death

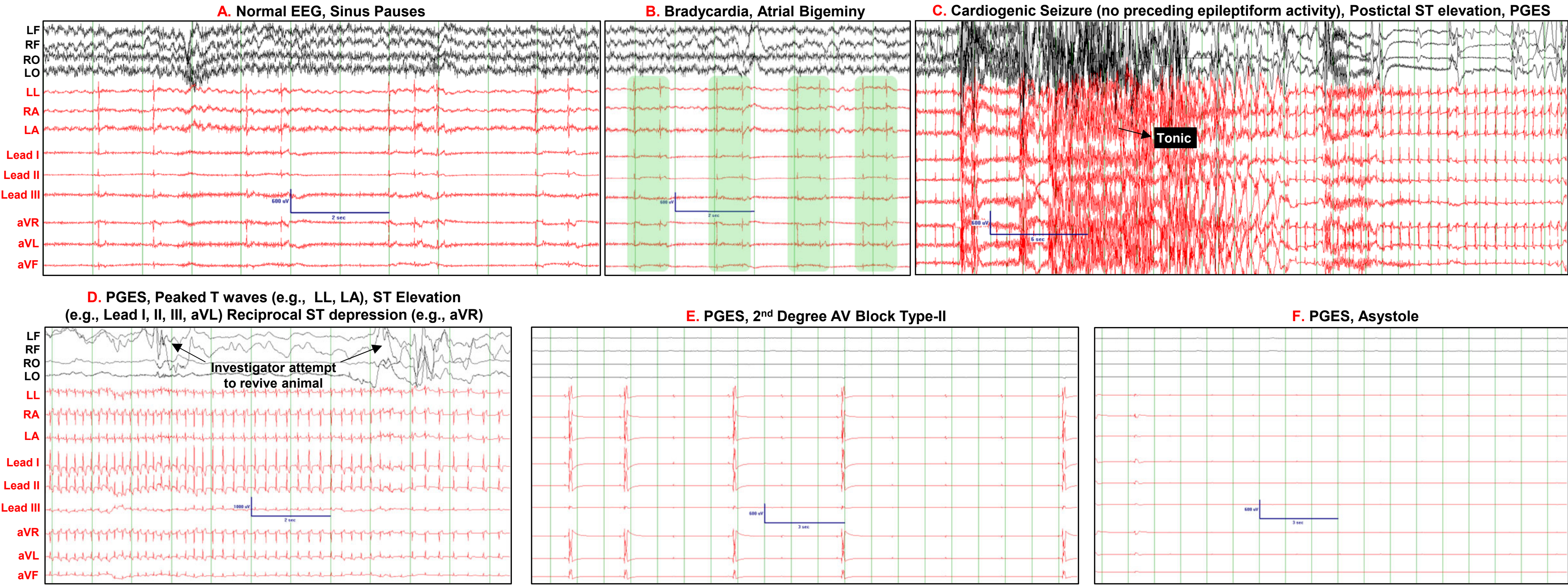

Supplementary Fig. 10: Cardiac abnormalities noted preceding lethal event (SCD) in Sudden death Case 2: *Kcnh2*$^{(+/7bp\text{-}del)}$ male 7-week-old rabbit. All depicted using referential EEG, referential ECG, and standard (I, II, III, and augmented) ECG lead configurations. **A.** EEG devoid of abnormal epileptic activity. ECG depicts sinus pauses (~1.5–2 sec). **B.** Extreme bradycardia (60 bpm) and atrial bigeminy (highlighted in green). **C.** Cardiogenic seizure: no epileptiform activity precedes sudden onset motor clonic and tonic seizure activity and post-ictal generalized EEG suppression (PGES); post-ictal ST elevation and reciprocal ST depression. **D.** Peaked T waves in referential leads (LA, LL). ST elevation in leads I, II, and aVL and reciprocal ST depression in aVR. **E.** Extreme PGES and 2nd degree AV block type-2. **F.** Asystole. ECG scale bar indicates 600μV amplitude (A, B, E, F), 1000μV amplitude (D) and 2 seconds. EEG scale: 140μV amplitude.
